# Effect of sleep position in term healthy newborns on sudden infant death syndrome and other infant outcomes: A systematic review

**DOI:** 10.7189/jogh.12.12001

**Published:** 2022-07-16

**Authors:** Mayank Priyadarshi, Bharathi Balachander, Mari J Sankar

**Affiliations:** 1 Department of Neonatology, All India Institute of Medical Sciences, Rishikesh, Uttarakhand, India; 2 Department of Neonatology, St. Johns Medical College Hospital, Bangalore, Karnataka, India; 3 Department of Pediatrics, All India Institute of Medical Sciences, New Delhi, India

## Abstract

**Background:**

Though recommended by numerous guidelines, adherence to supine sleep position during the first year of life is variable across the globe.

**Methods:**

This systematic review of randomized trials and observational studies assessed the effect of the supine compared to non-supine (prone or side) sleep position on healthy newborns. Key outcomes were neonatal mortality, sudden infant death syndrome (SIDS), sudden unexpected death in infancy (SUDI), acute life-threatening event (ALTE), neurodevelopment, and positional plagiocephaly. We searched MEDLINE via PubMed, Cochrane CENTRAL, EMBASE, and CINAHL (updated till November 2021). Two authors separately evaluated the risk of bias, extracted data, and synthesised effect estimates using relative risk (RR) or odds ratio (OR). The GRADE approach was used to assess the certainty of evidence.

**Results:**

We included 54 studies (43 observational studies and 11 intervention trials) involving 474 672 participants. A single study meeting the inclusion criteria suggested that the supine sleep position might reduce the risk of SUDI (0-1 year; OR = 0.39, 95% confidence interval (CI) = 0.23-0.65; 384 infants), compared to non-supine position. Supine sleep position might reduce the risk of SIDS (0-1 year; OR = 0.51, 95% CI = 0.42-0.61; 26 studies, 59332 infants) and unexplained SIDS/severe ALTE (neonatal period; OR = 0.16, 95% CI = 0.03-0.82; 1 study, 119 newborns), but the evidence was very uncertain. Supine sleep position probably increased the odds of being 0.5 standard deviation (SD) below mean on Gross Motor Scale at 6 months (OR = 1.67, 95% CI = 1.22-2.27; 1 study, 2097 participants), but might have little to no effect at 18 months of age (OR = 1.16, 95% CI = 0.96, 1.43; 1 study, 1919 participants). An increase in positional plagiocephaly at 2-7 months of age with supine sleep position is possible (OR = 2.77, 95% CI = 2.06-3.72; 6 studies, 1774 participants).

**Conclusions:**

Low- to very low-certainty evidence suggests that supine sleep position may reduce the risk of SUDI (0-1 year) and SIDS (0-1 year). Limited evidence suggests that supine sleeping probably delays short-term ‘gross motor’ development at 6 months, but the effect on long-term neurodevelopment at 18 months may be negligible.

Sudden infant death syndrome (SIDS) is defined as “the sudden unexpected death of an infant <1 year of age, with onset of the fatal episode apparently occurring during sleep, that remains unexplained after a thorough investigation, including performance of a complete autopsy and review of the circumstances of death and the clinical history” [1]. Sudden unexpected death in infancy (SUDI) is a broader term, which includes all sudden and unexpected deaths in infancy, unexplained (SIDS) or explained (suffocation, malformations, arrhythmias, etc). The incidence of SIDS peaks between the ages of 1 and 4 months, with 90% cases occurring before 6 months of age. A triple risk model has been proposed for the pathogenesis of SIDS, which requires the convergence of three risks: exogenous stressors (prone sleep, soft bedding etc), critical development period (2-4 months of age), and vulnerable infant (preterm, low birth weight, nicotine exposed etc) [2].

Prone and side sleep positions have been considered as external stressors for infants, based on observational studies, and supported by numerous physiological studies. These studies have shown that prone sleep position may alter the autonomic control of the infant cardiovascular system, particularly at 2 to 3 months of age, and may result in decreased cerebral oxygenation [3,4]. Prone position was also shown to decrease cardiac output, mean arterial pressure, oxygen saturation, minute ventilation, and arousal responses to various stimuli [5-8].

The initial safe sleep recommendations were published in the early 1990s after a realization in the late 1980s that prone sleep position was strongly associated with SIDS [9]. The launch of safe sleep campaigns was followed by a significant decline in the rates of SIDS and SUDI in the United States and other parts of the world [10]. The American Academy of Pediatrics (AAP) latest 2016 guidelines recommend placing every infant in supine position for every sleep by every caregiver until one year of age [11]. However, some studies have raised concerns about prolonged supine positions causing delayed motor development and deformational plagiocephaly [12,13].

Even several years after “Back to Sleep” campaign, some parents continue to place their newborns and infants in non-supine position, thus putting them in a potentially unsafe environment [14]. Therefore, it is necessary to look systematically into evidence and further strengthen the recommendations for a safe sleeping position for neonates and infants. This review aimed to determine the effect of supine sleep position compared to non-supine sleep position on health outcomes in term healthy newborns and infants (neurodevelopment, plagiocephaly, SIDS, SUDI, and acute life-threatening events (ALTE)).

## METHODS

Randomized controlled trials (RCTs) including cluster randomized trials or quasi-randomized trials in human neonates were eligible for this review. If the number of RCTs was found to be inadequate (<3) or the optimal information size was not met, we included the observational studies (before-after/cohort/case-control/cross-sectional analysed like case-control). The study population considered were term neonates (up to 28 completed days of life). We excluded the studies if most participants (≥50%) either had low birth weight or were preterm neonates. Studies were included if supine sleep position was compared with non-supine (prone or side) sleep position in neonates. Studies that did not report the number of infants in supine sleep position separately were excluded.

The outcomes of interest were: neonatal mortality (all-cause death in the first 28 days of life); SIDS; ALTE (an episode that is characterized by some combination of apnoea, colour change, marked change in muscle tone, choking or gagging) [15]; positional plagiocephaly (flattening of the skull) and physiological parameters (cerebral regional oxygen saturation, cardiac output/stroke volume etc.).

With inclusion criteria described above, only one of the included studies reported the risk of SIDS in the neonatal period while none reported the risk of neonatal mortality – the two critical outcomes of the review [16]. Therefore, we modified our inclusion criteria to include all studies reporting SIDS for infants (up to 1-year age). We included additional outcomes which were considered critical to this review: SUDI and long-term neurodevelopment (as assessed by standardized/ validated neurodevelopment tools).

### Search methodology

We initially searched for the existing systematic reviews. We planned to update the existing reviews depending upon the year of publication: if published in 2019 or 2020, we intended to use the results of the review without updating them; if published before 2019, we planned to update the review using the same search strategy used in the review.

The databases were searched independently by two authors (MP and BB). The search was conducted in the following databases: MEDLINE (1966 onwards) via PubMed, Cochrane Central Register of Controlled Trials (CENTRAL, The Cochrane Library), EMBASE (1988 onwards), and CINAHL (1981 onwards). We conducted the first search by March 31, 2020 and updated it until November 30, 2021. Searches were limited to human studies. There were no language restrictions. Related conference proceedings (like Pediatric Academic Societies (PAS) abstracts) were also be searched for relevant abstracts. Organizations and researchers in the field were contacted, if necessary, for information on unpublished and ongoing trials. Reference lists of all relevant studies were searched. The ClinicalTrials registry (www.clinicaltrials.gov) was searched to identify any ongoing trial. The search strategy is provided in Appendix S1 in **Online Supplementary Document**.

### Data extraction and management

Two authors (MP and BB) extracted data independently using a pilot-tested data collection form to collect information on design, methods, participants, interventions, outcomes, and treatment effects from each included study. We discussed disagreements until we reached a consensus. If data from trial reports were insufficient, we contacted study authors to request further required information or clarifications. Data were extracted from a systematic review (Gilbert 2005) for six studies (full text unavailable) [9]. Additional data were obtained by contacting the review author.

### Assessment of risk of bias in included studies

Two authors (MP and BB) independently assessed the methodological quality of the selected studies. Quality assessment was undertaken using the Cochrane Risk of Bias (RoB 2.0) tool for randomized trials and the ROBINS-I tool for observational studies [17,18]. Any disagreements between the review authors were resolved by discussion.

### Statistical analysis

Meta-analysis was performed with user-written programs on Stata 15.1 (StataCorp, College Station, TX, USA). Pooled estimates for categorical outcomes were calculated from the relative risk (RR) or odds ratio (OR) and 95% confidence intervals by the generic inverse variance method. If available, we used the adjusted RR and OR from the studies for pooling the results in the meta-analysis. The studies recorded different infant sleep positions such as last sleep position, usual sleep position, and position last found at the death scene. The last sleep position was used (or usual sleep position, if the former was not reported) for estimating the odds ratios in individual studies, if more than one position was reported. The studies reported sleep positions as supine, prone, and side. We considered prone and side position as non-supine and compared them with supine position as the side position has also been shown to increase the risk of SIDS in a previous systematic review [9]. We examined heterogeneity between study results by inspecting the forest plots and quantifying the impact of heterogeneity using the *I^2^* statistic. If there was no significant heterogeneity (*I^2^*<60% or *P* ≥ 0.1), we pooled the results using the fixed-effect model. If there was significant heterogeneity (*I^2^*>60% or *P* < 0.1), we explored the possible causes of heterogeneity. If there was no obvious clinical heterogeneity, we used the random-effects model for meta-analysis. The possibility of publication bias was evaluated using funnel plots and the Egger and Begg tests. We used GRADEpro software for assigning the certainty of evidence [19].

## RESULTS

**Figure 1:**
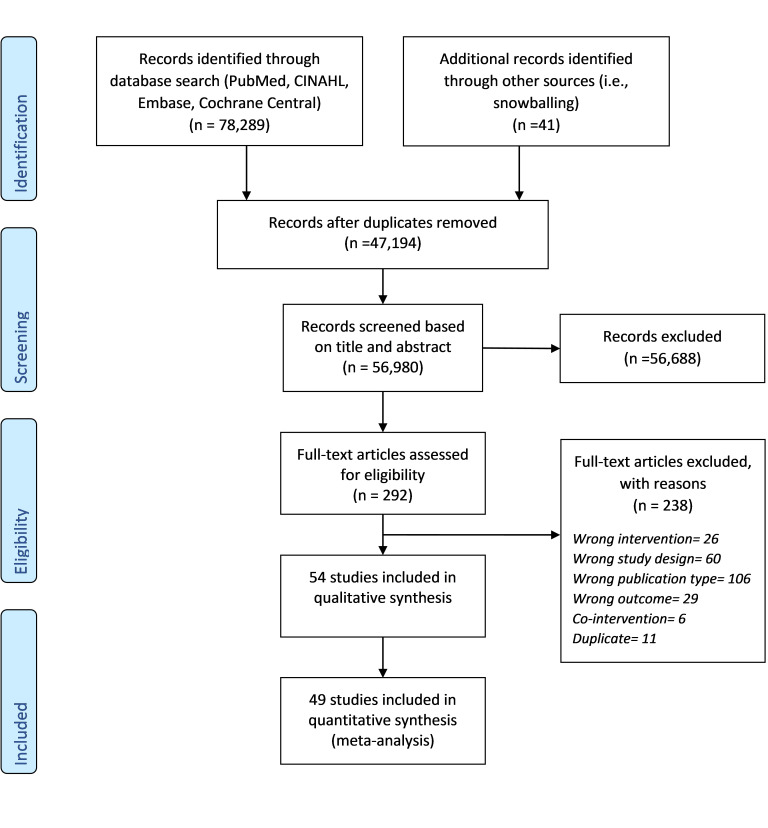
PRISMA flow chart depicting the selection of studies included in the review.

We found one systematic review assessing associations between infant sleeping positions and SIDS, which included observational studies published till January 2003 [9]. We performed a further search using a date filter from July 2002 until November 2021 (**Figure 1**). We included 54 studies, of which 49 studies were included in the quantitative analysis (**Table 1**).

**Table 1:** Characteristics of the studies included in the review

S.No.	Study author, year	Setting (level) / LMIC or HIC	Study design	Study population/Mean BW/gestation	Case (observational studies)/Intervention (trials) details	Control (observational studies/trials) details	Outcome parameters of interest	Results	Comments
1.	Arnestad 2001 [20]	Community, Southeast Norway; HIC	Case-control study.	N = 549 (cases = 174, controls = 375). Gest/BW not mentioned.	Cases of SIDS during the period 1984-1998 in Southeast Norway (death between second week after birth and 3 y of age included as SIDS deaths) Median age = 3 mo.	Controls: 375 age and sex-matched controls in southeast Norway. Data collected by questionnaire distributed by mail in 1993 and 1998.	Changes in risk factors for SIDS after decrease and stabilization of SIDS rate.	After 1993, maternal smoking during pregnancy, young maternal age, and co-sleeping became significant risk factors for SIDS.	Multivariate analysis used; serious risk of bias due to recall bias as mean time between death and completing the questionnaire was three years (range 1-8 y); 7.7% of the population were born at <37 weeks.
2.	Ballardini 2018 [13]	Public immunization clinic in Ferrara, Italy; HIC	Prospective cohort study.	All term healthy infants presenting at the public immunization clinic in Ferrara at 8 to 12 weeks of age. Exclusion criteria: cranio-synostosis, malformations, neurological diseases, or admitted to the neonatal intensive care unit. N = 283 (cases = 107, controls = 176). Gest = 39.3wk/BW = 3.3 kg.	Infants with plagiocephaly assessed by Argenta’s assessment tool Age at evaluation- 8-12 weeks.	Infants without plagiocephaly Age at evaluation = 8-12 weeks.	Risk factors for plagiocephaly **Risk factor of interest**: infant sleeping position (noted at 8-12 weeks at the time of visit).	Significant risk factors: lower head circumference, advanced maternal age, Italian compared to African, supine sleep position, preference for one side of the head. OR (prone vs supine) = 0.13 (95% CI = 0.03-0.40); OR (side vs supine) = 0.22 (95% CI = 0.05-0.71).	Univariate analysis used (confounding not addressed); sleep position noted at 8-12 weeks; use of Argenta’s criteria, which does not provide objective measures.
3.	Beal 1986 [21]	Community, Australia; HIC	Case-control study.	N = 285 (cases = 133, controls = 152) Gest/BW not mentioned.	Cases of SIDS in South Australia from 1970-1984 interviewed within weeks of death by Beal Mean age- not available.	Controls: postal survey of 200 consecutive birth registrations in August 1984 Age at evaluation- not available.	Association of sleep position with SIDS	Lower incidence of SIDS in communities that invariably use the supine sleeping position for infants than in those who do not OR (prone vs supine) = 6.71 (95% CI = 2.97-14.49).	Full text not available, data extracted from another study [9]; old study (significant improvement in medical care since the study period); univariate analysis used.
4.	Blabey 2009 [22]	Community, USA; HIC	Case-control.	N = 26 942 (cases = 88, controls = 26 854). Gest/BW not mentioned.	Cases of SIDS that occurred while bed sharing during 1992-2004 (Data collection from case files) Mean age- 3 mo.	Controls: Data from Pregnancy Risk Assessment Monitoring System (PRAMS) among all live births in Alaska during 1996–2003. (Data collection from mail or phone survey).	Prevalence of risk factors for SIDS while bed sharing.	Almost all bed-sharing deaths occurred in association with other risk factors (maternal tobacco use, sleeping with an impaired person etc).	Univariate analysis used; data used from all bed sharing cases and controls.
5.	Blair 2014 [23]	Community, UK; HIC	Case-control.	N = 1774 (cases = 392, controls = 1382). Gest/BW not mentioned (<37 wk = 148, <2500 g = 155).	Cases of SIDS from two case-control studies in UK during 1993-1995, and 2003-2006 Mean age = 98 d.	Controls: matched by age and area from same health visitor list as case. Data collected at home interview in both groups.	Quantify whether there is a risk of SIDS associated with co-sleeping in the absence of known hazards and explore the interactions with other known significant predictors of SIDS	The risk associated with bed-sharing in the absence of hazards was not significant overall (OR = 1.1, 95% CI = 0.6-2.0), for infants less than 3 mo old (OR = 1.6 95% CI = 0.96-2.7), and was in the direction of protection for older infants (OR = 0.1, 95% CI = 0.01-0.5).	Univariate analysis used; 8.3% of study population were <37 weeks; data for SIDS cases presented in study (original studies done on SUDI)
6.	Brooke 1997 [24]	Community, Scotland; HIC	Case-control.	N = 389 (cases = 133, controls = 256). Gest/BW not mentioned.	All cases of SIDS (infants aged 1 week to 1 y) in Scotland during 1992-1995 Mean age = 15 weeks.	Controls: matched for age, time, and same maternity unit. Data collected at home visits for both groups.	To investigate the relation between routine infant care practices and SIDS in Scotland.	Sleeping prone and parental smoking confirmed as modifiable risk factors for SIDS.	Multivariable analysis used; home visits within 3 weeks of death to limit recall bias.
7.	Bubnaitiene 2005 [25]	Community, Lithuania; HIC	Case-control.	N = 180 (cases = 35, controls = 145). Gest/BW not mentioned (<37 wk = 5, <2500 g = 7).	35 cases of infants who died from SIDS during the period of 1997-2000. Data collected by interview during home visit after 4 y. Mean age = 114 d.	Controls: 145 control infants matched with SIDS infants for date of birth and for region of birth. Questionnaires mailed to parents.	To identify risk factors for SIDS in Lithuania.	No significant association of sleeping positions with SIDS (possibly due to rare prone sleeping and more prevalent side than supine sleeping in the controls as well as the cases).	Univariate analysis used; strong recall bias likely in cases.
8.	Carpenter 1965 [26]	Community, UK; HIC	Case-control	N = 290 (cases = 107, controls = 183). Gest/BW not mentioned.	Cases of SIDS during 1958-1961 referred to coroner in 12 London boroughs (aged 2 weeks-2 y; six SIDS victims were aged >12 mo). Position found recorded by coroner. Mean age = 4.3 mo.	Controls: matched for age, sex, and community from register of Medical Officer of Health. Sleeping position recorded by health visitors.	Association of infection, suffocations and bottle-feeding in cot death (SIDS).	SIDS associated with a history of respiratory symptoms, use of soft pillows and mattresses, mouth and nose covered by bedding, and a history of early bottle-feeding.	Full text not available, data extracted from another study [9]; old study; univariate analysis used.
9.	Carpenter 2013 [27]	Community, UK, Europe, and Australasia; HIC	Case-control	N = 6151 (cases = 1 472, controls = 4679). Gest/BW not mentioned (<2500 g = 450).	Cases of SIDS from 5 studies- the European case-control studies 1992–1996, ie, European Concerted Action on SIDS (ECAS), the Scottish 1996–2000, the New Zealand 1987–1990, the Irish 1994–2003 and the German GeSID 1998–2001 data sets Mean age = 7-10 weeks.	Controls: living control infants randomly selected of similar age, time, and place	To ascertain the risk of SIDS associated with sleeping in bed with baby if neither parent smokes and the baby is breastfed.	Bed sharing for sleep when the parents do not smoke or take alcohol or drugs increases the risk of SIDS.	Univariate analysis used; 7.3% of study population were <2500 g
10.	Davis BE 1998 [28]	Medical university f/b community, USA; HIC	Prospective cohort study.	Full term infants recruited before the age of 2 mo Exclusion: gestational age <37 weeks; orthopaedic problems that might affect motor development; hyperbilirubinemia requiring hospitalization; any genetic or metabolic abnormalities; and asymmetric neurologic examinations. N = 351 (supine = 97, prone = 57). Gest not mentioned/BW = 3.5 kg.	Prone sleepers: Position noted from 1st week-5 mo; regular assessment with age by parents; consistent sleepers defined as adherence of at least 70% of the time.	Supine sleepers.	Age of acquisition of eight motor milestones (last milestone to be achieved among these: walking independently), assessed by monthly telephonic contact and 12-mo physician visit.	Prone sleepers attain several motor milestones earlier than supine sleepers (rolling prone to supine, tripod sitting, creeping, crawling, and pulling to stand); but all infants attained milestones within the accepted time range for normal.	Data presented only for consistent sleepers (154 infants from a total of 351); results may be limited by the accuracy of the parents’ responses.
11.	Dewey 1998 [29]	Medical college f/b community, UK; HIC	Prospective cohort study.	Infants, delivered during 1991-1992, followed to 18 mo of age N = 14 138 live births; 12 208 at 4 wk; 10579 at age 25-42 weeks, and 10 183 at 18-22 mo. Gest/BW not mentioned.	Sleeping position at 4-6 weeks, 6 mo, and 18 mo of age (assessed through questionnaire) Prone = 466 (4 wk); 382 (6 m); 384 (18 m) Side = 8316 (4 wk); 7212 (6 m); 7032 (18 m).	Supine = 2381 (4 wk); 2069 (6 m); 1915 (18 m) Variable = 1045 (4 wk); 916 (6 m); 852 (18 m).	Social, communication, fine and gross motor, and total developmental scales based on Denver Developmental Screening Test at 6 and 18 mo.	At 6 mo of age, infants put to sleep prone had a mean score 0.38 SD higher on the gross motor scale, 0.11 SD higher in the social skills scale, and a total development score 0.20 SD higher than those on their backs. No differences at 18 mo.	Multiple linear regression used to adjust for confounders; consistent sleeping position in the study population (only 4% difference in sleep position at 4 wk, 6 mo and 18 mo).
12.	Dwyer 1999 [30]	Community, Tasmania, Australia; HIC	Nested case-control in a prospective study.	Eligible infants were one-fifth of Tasmanian live births, assessed to be in highest scoring quintile of a perinatal risk score for SIDS Exclusion: severe neonatal disease, major congenital anomaly and infants for adoption. N = 9655 (cases = 37, controls = 9618). Gest/BW not mentioned.	Cases of SIDS from the study cohort during 1988-1995, measuring usual sleeping position at 1 mo of age. Median age = 14 weeks.	Controls: comparison cohort selected as highest scoring quintile using at risk score for SIDS. Includes data from Dwyer 1991 [31].	Relation between sleeping position and morbidity at 1 and 3 mo, post neonatal mortality and SIDS.	Supine position at 1 mo was not associated with any increase in short-term morbidity or post neonatal mortality.	Univariate analysis used; all infants at risk for SIDS according to perinatal scoring system; infant position at 1 mo used for analysis (issue of non-adherence likely).
13.	Engelberts 1991 [32]	Community, Netherlands; HIC	Case-control.	N = 671 (cases = 105, controls = 566). Gest/BW not mentioned.	Cases of SIDS in Netherlands during 1987-89. Data collection by parent-completed postal questionnaire after telephone contact. Mean age not mentioned.	Controls: randomly selected from municipal registers. Data collection by postal questionnaire asking about usual sleeping position in each of months 1–6. Data for month 3 used in analyses.	Epidemiological study of cot deaths (SIDS) in Netherlands.	No significant association of sleeping position with SIDS found.	Full text not available, data extracted from another study [9]; old study; univariate analysis used.
14.	Fister 2020 [33]	UniversityMedical Centre, Slovenia; HIC	Prospective intervention study (crossover).	Term hemodynamically stable neonates (mean age = 11 d) Exclusion: Preterm, HIE, infection, congenital abnormalities. N = 46. Gest = 39 wk, BW = 3.4 kg.	After feeding, sleeping newborns put in supine position with a 30° head-up tilt of the bed for 30 min. ECG signal recorded in four positions: 1. Prone with tilt 2. Prone without tilt.	3. Supine with tilt 4. Supine without tilt.	Parameters of HRV (heart rate variability) as assessed by ECG signals.	In term newborns, sleeping position associated with HRV. Better autonomic stability found in supine position.	Lack of electroencephalographic data of the sleep stages.
15.	Fleming 1991 [34]	Community, UK; HIC	Case-control.	N = 198 (cases = 67, controls = 131). Gest/BW not mentioned.	Cases of SIDS in Avon, UK, during 1987-1989 interviewed at home within 3 d of death. Mean age = 94 d.	Controls: matched by age and area based on same health visitor list as SIDS victim. Data collection at home interview for both groups.	To determine the relation between sleeping position and quantity of bedding and SIDS.	Overheating and prone position independently associated with an increased risk of SIDS, particularly in infants aged >70 d.	Univariate analysis used; old study.
16.	Froggat 1970 [35]	Community, Ireland; HIC	Case-control.	N = 282 (cases = 139, controls = 143). Gest/BW not mentioned.	Consecutive cases of SIDS during 1965-1967 in Northern Ireland Median age = 14 weeks.	Controls: matched for age, sex, and administrative area. Data collection by home interviews in both cases and controls.	Epidemiological study of cot deaths (SIDS) in Ireland.	Prone sleeping significantly associated with SIDS compared to supine position.	Univariate analysis used; old study.
17.	Gormally 1994 [36]	Community, Ireland; HIC	Case-control.	N = 191 (cases = 93, controls = 98). Gest not mentioned/BW = 3.4 kg.	SIDS: cases identified by the Sudden Infant Death Association in Ireland. Mean age- 4 mo.	Controls: matched for sex and age from Rotunda Hospital records in Dublin. Data collection by postal questionnaires in both groups.	To compare the frequency of different sleeping positions in a group of SIDS and control infants.	Relative risk of SIDS of 2.3 comparing prone to side and 10.5 comparing prone to supine positions.	Univariate analysis used; the time period during which SIDS occurred not mentioned.
18.	Hauck 2002 [37]	Community, USA; HIC	Case-control.	N = 516 (cases = 258, controls = 258). Gest not mentioned/ BW = 2.8 kg.	SIDS: cases in Chicago Mean age = 89 d.	Controls: selected from the Chicago birth registry matched for maternal age, child’s age, and birth weight. Groups of 20-40 controls selected and those responding first included. Data collection at home interview for both cases and controls.	Risk of SIDS related to prone sleeping position adjusting for potential confounding variables and other risk factors for SIDS, and comparisons by race-ethnicity.	Prone sleeping was found to be a significant risk factor for SIDS in this primarily African American urban sample.	Multivariable analysis used; interview conducted within 2 weeks of death.
19.	Horne 2000 [7]; Richardson 2008 [38]; Tuladhar 2002 [39]; Tuladhar 2005 [40]	Medical university, Melbourne, Victoria, Australia; HIC	Randomized crossover trial.	Term infants with normal birth weight & Apgar scores. Exclusion: congenital abnormalities; N = 24 (11 infants born to mothers who smoked were excluded in one study [38]). Gest = 40 wk/BW = 3.5 kg.	Infants studied on 3 occasions: 2-3 weeks, 2-3 mo, and 5 to 6 mo of age. After the infant was in a stable sleep state, each infant slept in (at all time points): 1. Active sleep (prone) 2. Quiet sleep (prone).	3. Active sleep (supine) 4. Quiet sleep (supine).	Arousal from sleep, heart rate (HR) responses following provoked arousal, HR responses to non-arousing trigeminal stimulation, nature of both induced and spontaneous arousal responses.	Prone position significantly impairs arousal from both active sleep and quiet sleep, promotes full cortical activation at 2-3 mo of age, elevates basal heart rate and impairs heart rate control in term healthy infants.	Four articles on physiological responses published on the same study population.
20.	Hunt 2003 [41]	Community, USA; HIC	Prospective cohort study.	Study cohort from Infant Care Practices Study, who had consistent sleeping position at 1, 3 and 6 mo. N = 3729 (cases = 6, controls = 3723). Gest not mentioned/BW = 3.5 kg.	Cases: Hospital admissions related to Apparent Life-Threatening Events (ALTE) reported in study cohort in Massachusetts and Ohio (3733 infants) with sleep positions at ages 1, 3, and 6 mo. Follow-up: up to 6 mo.	Controls: from the same cohort. Data collected prospectively through mailed questionnaire or by telephone at ages 1, 3 and 6 mo.	Health outcomes in infants aged 1-6 mo in relation to sleep position (hospital admissions, fever, cough, respiratory problem, diarrhoea, stuffy nose etc).	No identified symptom or illness was significantly increased among non-prone sleepers during the first 6 mo of life.	No difference in hospital admissions due to ALTE between any sleeping position; univariate analysis used.
21.	Hutchison 2003 [42]	Hospital clinics, New Zealand; HIC	Case-control study.	N = 194 (cases = 100, controls = 94). Gest = 38-39 wk/BW = 3.1-3.4 kg.	Cases: Non-synostotic plagiocephaly (confirmed by visual and anthropometric examinations) Mean age- 25 weeks.	Controls: Every sixth infant aged 2-12 mo from database of Auckland region of the Plunket society (total = 200; respondents = 94) Mean age = 28 weeks.	Identify the determinants of non-synostotic plagiocephaly.	Risk factors: male, firstborn, preterm, sleep supine only, and born to less educated mothers.	Multivariate analysis used; recall bias very likely (sleep position at 6 weeks assessed at an age of 25-28 weeks); 12% infants <37 wk.
22.	Hutchison 2004 [43]	Community maternity unit, New Zealand; HIC	Prospective cohort study.	A cohort of infants born in North Shore Hospital, Auckland Exclusion: congenital deformities, not domiciled in the Waitemata Health District, those planning to move out of the region in the next year, and those not seen in the first week N = 200 (cases = 39, controls = 161). Gest/BW- not mentioned; <37 wk = 2%.	Cases: Cohort infants who developed non-synostotic plagiocephaly (diagnosed with photography and calculation of cranial length ratio and cephalic index). Mean age of assessment = 6 weeks and 4, 8, 12, and 24 mo.	Control: Cohort infants from the cohort who did not develop plagiocephaly Risk factors assessed during newborn period and at follow up visits. Mean age of assessment = 6 weeks and 4, 8, 12, and 24 mo.	Assessment of the prevalence and natural history of non-synostotic plagiocephaly (NSP) in normal infants in the first 2 y of life, and ascertainment of the risk factors at 6 weeks and 4 mo.	Prevalence of NSP at 6 weeks and 4, 8, 12, and 24 mo was 16.0%, 19.7%, 9.2%, 6.8%, and 3.3% respectively; limited head rotation, lower activity levels, and supine sleep position important determinants of NSP.	Univariate analysis used; odds ratio of newborn sleep position for development of NSP at 4 mo used in meta-analysis (NSP usually takes 2-3 mo to manifest, hence most NSP would have occurred by 4 mo); adherence to newborn sleep position was not mentioned at follow up visits.
23.	Hutchison 2009 [44]	Plagiocephaly clinic, New Zealand; HIC	Retrospective cohort study.	Infants attending the plagiocephaly clinic N = 285 (cases = 223, controls = 62). Gest/BW not mentioned.	Cases: Infants diagnosed with plagiocephaly or brachycephaly (diagnosed with photography and calculation of cranial length ratio and cephalic index) Mean age of assessment = 22 weeks (16-29 weeks).	Controls: Infants found to have normal head shape on evaluation. Risk factors (sleep position during first 6 weeks) assessed at the same visit Mean age of assessment = 22 weeks (16-29 weeks).	Characteristics, developmental status, and severity of head shape deformation in infants presenting to a plagiocephaly clinic.	Males, firstborn infants, instrument-delivered infants, supine sleep position and right-sided flattening were predominant among the cases.	Univariate analysis used; recall bias very likely (sleep position during first 6 weeks assessed at an age of 16-29 weeks); selection bias likely (controls were infants presenting with concerns of plagiocephaly at the clinic).
24.	Iyasu 2002 [45]	Community, USA; HIC	Case-control study.	N = 98 (cases = 33, controls = 65). Gest = 39 wk/BW = 3.3 kg.	Cases of SIDS among American Indians in South Dakota, North Dakota, Nebraska, and Iowa during 1992-1996. Mean age = 109 d.	Controls: 2 living controls among American Indians from the same regions matched for postnatal age and community. Data collected by parental interviews.	Risk factors for SIDS among northern plains Indians.	Risk factors: Public health nurse visits, maternal alcohol use during the periconceptional period and first trimester, and layers of clothing.	Univariate analysis used; small sample size; standard death scene forms not completed on all unattended deaths.
25.	Jonge 2005 [46]	Community, Netherlands; HIC	Descriptive national survey. (analysed like case-control).	N = 2725 (Cases = 190, controls = 2535). Gest/BW not mentioned.	Cases of SIDS among children 7-365 d in the years 1980-2004 were taken from the Central causes of death statistics Bureau of Statistics. Median age = 3 mo.	Controls: general population in infants (0-9 mo) were derived from studies under SIDS (1984-1991 and 1996-2004) and periodic surveys at health centres infants (1985-2004).	Incidence of SIDS and prevalence of risk factors from 1980-2004.	Decrease in the incidence of SIDS and the prevalence of known risk factors, emergence of new risk factors.	Data extracted only for period 1996-2004 due to non-availability of numbers in control group in prior years (prior data presented in another study [32]); univariate analysis.
26.	Jorch 1994 [47]	Community, Germany; HIC	Case-control study.	N = 852 (Cases = 94, controls = 758). Gest/ BW- not mentioned.	SIDS: cases in two districts in Germany during 1990-1992. Data collected at home interview. Median age not mentioned.	Controls: postal survey in two districts of representative sample in Autumn 1991.	Risk factors for SIDS	Prone sleep position a risk factor for SIDS OR (prone vs supine) = 6.85 (95% CI = 3.22-14.58).	Full text not available, data extracted from other study [9]; old study; univariate analysis used.
27.	Katz 2014 [48]	High-altitude community, Colorado, USA; HIC	Retrospective cohort study	N = 393 216. Gest not mentioned/BW = 3.2 kg	SIDS: infant death registries data over a 22-y period (1990-2012) provided by the Colorado Department of Public Health and Environment (CDPHE) Median age not mentioned.	Control: all infants born in Colorado, to mothers residing in Colorado, from 1990 to 2012.	Association between residential altitude and SIDS.	Residence at high altitude significantly associated with SIDS. Incidence of SIDS 1.99/1000 live births prior to back to sleep campaign and dropped to 0.57/1000 live births after its implementation.	Included in qualitative review only (numbers not available for different sleeping positions).
28.	Klonof-Cohen 1995 [49]	Community, USA; HIC	Case-control study	N = 383 (Cases = 193, controls = 190). Gest not mentioned/BW = 3.3 kg.	Cases of SIDS in five health departments in southern California during 1989-92. Median age = 2-4 mo.	Controls: matched by birth date, hospital of birth, sex, and race. Data collection in both groups by telephone interview before adverse publicity about sleeping position.	Association between different sleep positions and SIDS.	Routine prone sleep position was not associated with an increased risk of SIDS in this study population.	Univariate analysis used; Control interviews conducted 3-6 mo after case interviews.
29.	Lee 1988 [50]	Community, Hong Kong; HIC	Case-control study	N = 48 (Cases = 16, controls = 32). Gest not mentioned/BW = 3.3 kg.	Cases of SIDS during 1986–1987 prospective surveillance in Hong Kong. Data collected at home interview Mean age not mentioned.	Controls: age and sex matched, one from hospital and one from community. No details given on data collection.	Incidence of SIDS in Hong Kong.	Prone position a risk factor for SIDS.	Univariate analysis used; limited information (published as letter to editor).
30.	Leung 2017 [51]	Hospital, Australia; HIC	Prospective cohort study.	Full term infants Exclusion: APGAR scores of <7 at 1 min or 5 min, with an identified neurological insult; low birth weight (<2500g at term); or a diagnosed medical or orthopedic condition. N = 94. Gest not mentioned/BW = 3.3 kg.	Cases: Plagiocephaly measured at 9 weeks with the modified Cranial Vault Asymmetry Index.	Controls: Infants from study cohort who did not develop plagiocephaly.	Relationship between infant body and head positioning, with development of asymmetrical head orientation and/or positional plagiocephaly.	More severe plagiocephaly was associated with longer supine sleep – maximum (*P* = 0.001) and longer supine-lying-total (*P* = 0.014) at 6 weeks.	Individual numbers of cases and controls not mentioned; multivariable analysis used; positioning assessed at 3, 6, and 9 weeks based on parents’ recall of last 3 d (found significant at 6 weeks).
31.	Li 2003 [52]	Community, USA; HIC	Case-control study	N = 476 (Cases = 166, controls = 310). Gest not mentioned (<37 weeks = 62)/BW = 2.9 kg.	Cases: SIDS from 11 counties in California during 1997-2000 Mean age = 98 d.	Controls: ethnicity/race- and age-matched controls chosen from birth certificates in the same county. Data from in-person interviews.	Association between infant sleeping position and SIDS in an ethnically diverse US population.	Infants last put down to sleep in the prone or side position were at greater risk of SIDS than were infants last put down on their backs.	Multivariable analysis used; recall bias likely (interview 4 mo after event); 13% study population <37 wk.
32.	Lucchini 2015 [53]	Medical University, Italy; HIC	Prospective intervention study	Full term infants Exclusion: none mentioned. N = 60 newborns; 22 (1-mo old) infants. Gest = 39 wk/ BW not mentioned	Prone position (30 newborns and 7 one-month infants).	Supine position (30 newborns and 15 one-month infants).	Heart rate variability and respiration parameters acquired during 30-min evaluation in each position.	In the comparison between positions during sleep, no parameter proved to be able of distinguishing the two conditions.	Only *P*-values provided (not included in meta-analysis).
33.	Ma 2015 [54]	Hospital, USA; HIC	Prospective intervention study (crossover).	Hemodynamically stable NICU infants Exclusion: malformations, mechanical ventilation and small for gestation. N = 30 (9 preterm <35 wk). Gest = 37 wk/ BW = 2.7 kg.	After neonates became completely calm or fell asleep, data collection while in supine position.	Subjects were then placed in prone position and the data were collected again, followed by being repositioned back-to-supine position for the final data collection (Each period lasted for 10 min.)	Heart rate (HR), stroke volume (SV) and cardiac output (CO) by electrical velocimetry; skin blood flow (SBF) using Laser Doppler, systemic vascular resistance (SVR) index (mean blood pressure/CO).	Short-term prone positioning is associated with decreased SV, CO and SBF and increased calculated SVR index.	Significant measurement errors for SV and CO; small sample size; findings apply only to the cardiovascular effects of short-term prone positioning.
34.	Majnemer 2006 [55]	Community, Canada; HIC	Prospective cohort study.	Healthy typically developing white infants at 4- or 6-mo of age with consistent sleep position during first weeks of life and at recruitment. Exclusion: <38 wk gestation, non-English and non-French speaking, torticollis, documented prenatal or perinatal complications, attendance in daycare where positioning practices may be less consistent than in the home. N = 155 (prone sleepers = 34, supine sleepers = 121). Gest/BW not mentioned.	Prone sleepers (consistently placed in prone when put down to sleep; assessed by a questionnaire enquiring sleep positions at the time of recruitment and during the first weeks of life) 4-mo-olds (N = 12) and 6-mo-olds (N = 22).	Supine sleepers Assessed similarly as for prone sleepers 4-mo-olds (N = 71) and 6-mo-olds (N = 50).	Motor performance assessed with Alberta Infant Motor Scale (AIMS) and Peabody Developmental Motor Scale (PDMS) at recruitment; and using PDMS and Battelle Developmental Inventory at 15 mo of age.	No significant difference in total AIMS raw scores or PDMS quotients at 4 mo, better AIMS raw scores and PDMS gross motor quotient in prone sleepers at 6 mo, no significant difference in PDMS quotients or Battelle Developmental Inventory Age equivalents at 15 mo.	Small number of prone sleepers; recall bias likely (assessment of sleep position during first weeks of life at 4-6 mo).
35.	Mawji 2014 [56]	Immunization clinics, Canada; HIC	Retrospective cohort study.	Healthy full-term infants ranging from 7-12 weeks of age who presented for immunization. N = 440 (cases = 205, controls = 235). Gest/BW- not mentioned.	Cases: infants diagnosed as positional plagiocephaly using clinical criteria (Argenta’s 5-point scale). Mean age- 7-12 weeks.	Controls: Infants found to have normal head shape (assessed at same age as cases) Risk factors (sleep position at 6 weeks) assessed at the same visit	Determination of potential risk factors for developing positional plagiocephaly in infants seven to 12 weeks of age	Risk factors: right-sided head positional preference, left-sided head positional preference, supine sleep position, vacuum/forceps assisted delivery and male sex	Multivariable analysis used; risk factors assessed using parent-filled questionnaire; possible bias in measurement (if exposure status known)
36.	McGlashan 1989 [57]	Community, Tasmania, Australia; HIC	Case-control study.	N = 493 (cases = 164, controls = 329). Gest/BW- not mentioned.	Cases of SIDS notified by coroners in Tasmania during 1980-1986. Mean age- not mentioned.	Controls: matched for age, sex, and hospital of birth. Data collection at home interviews in both groups.	Epidemiology of SIDS deaths in Tasmania.	Risk factors: cigarette smoking by parents, prone sleeping position, density of persons within the home.	Univariate analysis used; old study.
37.	Mitchell 1997 [58]	Community, New Zealand; HIC	Nested case-control.	Data collected by community child health nurses on all infants born in New Zealand at initial contact and at 2 mo. N = 834 (cases = 63, controls = 771). Gest/ BW not mentioned.	Cases of post-neonatal SIDS in New Zealand during 1991-1993 (neonatal SIDS excluded). Mean age not mentioned.	Controls: randomly selected to be representative of all births. Data for both groups were extracted from routine records recorded by Plunket nurses at initial contact and at ~ 2 mo of age.	To identify the risk factors for SIDS following a national campaign to prevent SIDS.	Risk factors: prone and side sleeping positions, maternal smoking, and the joint exposure to bed sharing and maternal smoking.	Multivariable analysis used; prospectively collected data.
38.	Mitchell 1999 [59]	Community, New Zealand; HIC	Case-control.	N = 1972 (cases = 388, controls = 1584). Gest/ BW not mentioned.	Cases of SIDS deaths within areas covering 80% of births in New Zealand during 1987-1990 Mean age- 16 weeks.	Controls: randomly selected in proportion to hospital births in same areas and frequency matched for predicted age and season of cases. Home interviews for both groups measuring position placed at nominated sleep.	To examine if prone sleep position increases the risk of SIDS, particularly in infants not used to prone position.	Infants put to sleep in supine position were at the lowest risk of SIDS.	Multivariable analysis used.
39.	Mitchell 2007 [60]	Delivered at a Women’s hospital f/b community, New Zealand; HIC	Survey	Survey done in 278 infants (143 infants aged 6-8 weeks and 135 infants aged 3-4 mo to derive usual infant sleep position in 2005) N = 278 Gest/ BW- not mentioned.	Annual SIDS mortality in 2005 (several years after national campaign to prevent SIDS).	SIDS rate in 1992 (around the time of national campaign) Annual SIDS mortality obtained from New Zealand Health Information Service publications.	To determine change in prevalence of side sleeping position and to compare its prevalence with changes in SIDS mortality.	Proportion of infants sleeping supine increased substantially (from 24.4% in 1992 to 72.3% in 2005), and could account for the 39%-48% decrease in SIDS mortality.	Survey data extrapolated to derive population attributable risk; not included in meta-analysis.
40.	Mitchell 2012 [61]	Community, New Zealand; HIC	Ecological study.	Population-based data analysis.	Post-campaign period (after 1990).	Pre-campaign period (post 1990).	Number of lives saved each year attributable to campaign.	Change in infant sleep position (from prone to side, then to predominantly supine) has saved over 3000 lives.	Not included in meta-analysis; population-based study.
41.	Mitchell 2017 [62]	Community, New Zealand; HIC	Case-control study.	N = 384 (cases = 126, controls = 258). Gest not mentioned/ BW = 3.1 kg.	SUDI: born and domiciled in New Zealand, and aged 7-365 d (including SIDS) during 2012-2015. Mean age = 14 weeks.	Control: 649 controls unmatched, however randomly selected to age and ethnicity in proportion to cases. Data collected by interviews.	To identify modifiable risk factors for SUDI.	Combination of bed sharing and maternal smoking leads to a greatly increased risk of SUDI.	Multivariable analysis used.
42.	Muller-Nordhorn 2021 [63]	Population based data, USA; HIC	Ecological study.	Trends of SUDI rates and immunization from national databases Data from 1992-2015.	SUDI: mortality data from Centers for Disease Control and Prevention (CDC).	Data on infant sleep position from the Pregnancy Risk Assessment Monitoring System (PRAMS) study.	Time trends in SUDI and their association with immunization coverage.	SUID mortality decreasing, and inversely related to immunization coverage.	**Not included in meta-analysis;** population-based study.
43.	Pinho 2011 [64]	Community, Brazil; UMIC.	Case-control.	N = 225 (cases = 33, controls = 192). Gest- 8.3 mo/ BW- 2.6 kg.	Cases: SIDS in infants between 28 and 364 d of age who died in their usual sleep period during 2001-2003. Mean age = 3 mo.	Control: Live controls were selected among children who lived on the same streets as SIDS cases. Data collected from interviews within 10 d of death.	Epidemiological profile, risk factors for SIDS.	Risk factors: ethnicity, prematurity, low birth weight, adolescent mother, smoking during pregnancy and family income below the minimum wage.	Univariate analysis used; data from developing country; prevalence of prone position very low in population.
44.	Poets 2009 [65]	University Hospital, Tuebingen, Germany; HIC	Randomized crossover trial.	Clinically well term neonates (0-5 d) admitted to the maternity or neonatal unit for common neonatal problems Exclusion criteria: none mentioned. N = 609 (476 recording pairs fulfilled study criteria). Gest = 39 wk/BW = 3.3 kg.	Side position: Infants were placed in a cotton sleeping bag with long sleeves in horizontal supine and side position in random sequence for 6 h each; no pillows were used.	Supine position.	Rate of desaturation events (<80%/h).	Adjusted OR for at least one desaturation event (supine vs side) = 2.0 (95% CI = 1.3-3.1).	No information on allocation concealment or deviation/adherence to protocol; data skewed, hence data dichotomized to give OR.
45.	Poets 2012 [16]	Hospital based record. Germany; HIC	Case-control.	Term infants with a 10-min Apgar score ≥8 (data from Surveillance Unit for Rare Pediatric Conditions). N = 119 (cases = 29, controls = 90). Gest = 39 wk/ BW not mentioned.	Cases: unexplained SID or Severe-ALTE within 24 h of birth (S-ALTE was defined as an acute state of cyanosis or pallor and unconsciousness, which was felt to require bagging, or intubation with or without cardiac compressions) during 2009-2010. Mean age- 90 min.	Control: three (near-)term infants with a 10-min Apgar score ≥8 born within a few days of event.	To identify potential risk factors for unexplained sudden infant deaths (SID) and severe apparent life-threatening events (S-ALTE) within 24 h of birth.	Risk factors: primipara (OR = 6.22; 95% CI = 2.11-18.32) and potentially asphyxiating position (OR = 6.45; 95% CI 1.22-34.10).	Univariate analysis used; potentially asphyxiating position refers to infant lying on mother’s breast (prone)/ abdomen or near to and facing her (side).
46.	Rossor 2018 [8]	College Hospital, UK; HIC	Randomized crossover trial.	Infants born at 36-42 wk gestation. Exclusion: major congenital abnormalities, respiratory disease, or sepsis. N = 22 (data from only control group as mothers smoked or misused drugs in other groups). Gest = 39 wk/BW = 3 kg	Prone position (After a feed, the infant was placed in either the prone or supine sleeping position, the other position being studied afterwards on the same day).	Supine position (hypoxic challenge- by providing containing 15% oxygen in nitrogen as inspired gas delivered from a cylinder).	Effect of maternal smoking, substance misuse and sleeping position on the newborn response to hypoxia (Minute ventilation (MV) and end-tidal carbon dioxide (ETCO2) levels).	In the controls, sleeping position had no effect on baseline ventilatory variables. The rate of decline in MV during hypoxia was greater in the supine compared to the prone position (*P* = 0.02).	No information on allocation concealment or baseline imbalances; small sample size.
47.	Tonkin 1986 [66]	Community, New Zealand; HIC	Case-control study.	N = 2073 (cases = 91, controls = 1982). Gest/BW not mentioned.	Cases of SIDS in Auckland, New Zealand (position found routinely recorded in 1972, 1973, and 1982).	Controls: Plunket nurses (health visitors) in Auckland noted sleeping position of 50 babies most recently seen (10 nurses in 1972, 15 nurses in 1973). In 1982 all nurses noted sleeping position of 2-week-old babies during a 3-mo period.	Epidemiology of cot deaths in Auckland.	Unadjusted OR for SIDS: Prone vs supine = 0.97 (95% CI = 0.44-2.11); Side vs supine = 0.27 (95% CI = 0.12-0.60).	Full text not available, data extracted from another study [9]; old study; univariate analysis used.
48.	Tonkin 1989 [67]	Community, New Zealand; HIC	Case-control study.	N = 1264 (cases = 126, controls = 1138). Gest/BW not mentioned.	Cases of SIDS in Auckland, New Zealand during 1981-1985. Data collected at interview.	Controls: surveyed by Plunket nurses in Auckland in 1983 aged 1-4 mo. Results used for usual position at 3 mo.	To examine association between infant sleeping position and cot death.	Unadjusted OR for SIDS: Prone vs supine = 0.69 (95% CI = 0.39-1.25); Side vs supine = 0.53 (95% CI = 0.28-0.98).	Full text not available, data extracted from another study [9]; old study; univariate analysis used.
49.	van Vlimmeren 2007 [68]	District hospital, Netherlands; HIC	Prospective cohort study.	Cohort of newborns >36 wk gestation without any dimorphisms or syndromes (congenital muscular torticollis excluded). N = 380. (cases = 84, controls = 296). Gest = 39 wk/BW = 3.4 kg	Cases: Infants diagnosed with deformational plagiocephaly at 7 weeks (using Plagiocephalometry (skull anthropometry) and diameter difference index >104%	Controls: Infants found to have normal head shape at 7-week assessment Risk factors noted at birth and follow up at 7 weeks.	Identification of risk factors for deformational plagiocephaly within 48 h of birth and at 7 weeks of age.	Risk factors: gender, birth rank, head position when sleeping, position on chest of drawers, method of feeding, positioning during bottle-feeding, and tummy time when awake.	Univariate analysis used; ‘sleep position after 2 weeks’ used as a putative risk factor (no details on adherence to sleep position).
50.	Wong 2010 [4]	Monash University, Melbourne, Australia; HIC	Randomized crossover trial.	Healthy term infants born to nonsmoking mothers, routinely slept in a supine position at home, and were breast fed. N = 17. Gest = 38-42 wk/BW = 3.7 kg.	Prone position (active sleep and quiet sleep) (infants studied on 3 occasions: 2-4 weeks, 2-3 mo, and 5-6 mo of age).	Supine position (active sleep and quiet sleep) (Daytime polysomnography to record the state of sleep).	Effects of sleeping position, sleep state, and postnatal age on cerebral oxygenation (tissue oxygenation index; TOI).	In infants who slept in the prone position, TOI was lower in both quiet sleep (QS) and active sleep (AS) at age 2 to 4 weeks and in QS at age 2 to 3 mo (*P* < 5).	No information on allocation concealment or baseline imbalances; small sample size.
51.	Wong 2019 [6]	Hospital. Taiwan; HIC	Prospective intervention study (crossover).	Healthy neonates (aged 2–3 d) born via vaginal delivery after an uneventful pregnancy Exclusion: prematurity, low birth weight, severe perinatal complications; babies with heavy parental smoking or maternal drug addiction. N = 17. Gest = 37-40 wk/BW = 3.2 kg.	Prone sleep position from 12 pm to 4 pm (Interrupted by a midday feeding).	Supine sleep position from 8 am to 12 pm (Daytime polysomnography to record the state of sleep).	Heart rate (HR), oxygen saturation, carbon dioxide concentration, sleep stages, central apnea index (CAI), obstructive apnea/ hypopnea index (OAHI), and oxygen nadir.	During prone sleep, neonates had a faster HR, decreased oxygen saturation, and a longer duration of oxygen saturation <90% than during supine sleep.	Lack of data regarding normal sleep respiratory parameters for neonates.
52.	Wu 2017 [69]	Hospital. Taiwan; HIC	Prospective intervention study (crossover).	Healthy term infants within first week of life Exclusion: congenital anomalies, patent ductus arteriosus, small for gestational age status. N = 34. Gest not mentioned/BW = 3.2 kg	Prone position (PP). Infants placed in supine (SP1), prone (PP) and back in supine (SP2) position for 15 min each while asleep.	Supine positions (SP1 and SP2) (Monitoring only when the infant was in the sleep state as determined by eye closure and lack of movement or reaction to echo).	Cardiac output (CO) and stroke volume (SV) assessed by electrical velocimetry (EV) and echo, and cerebral regional oxygen saturation (CrSO2) in the frontal lobes, heart rate (HR) and SpO2.	CO decreases in prone position due to a decrease in SV and not HR. CO recovers when placed back in supine.	No assessment of duration of the decrease in CO and SV during PP; no systematic assessment of sleep state of infants by polysomnography; imprecision of non-invasive methods in determining CO.
53.	Yiallourou 2008a [3]; Yiallourou 2008b [5]	Monash university, Australia; HIC	Randomized crossover trial.	Full-term infants at 38–42 weeks with Apgar scores of 9-10 at 5 min without congenital abnormalities, non-smoking mothers and routinely slept supine at home. N = 20. Gest not mentioned/ BW = 3.6 kg.	Prone position (active sleep and quiet sleep) (Head up tilts (HUT) of 15° were performed during active sleep (AS) and quiet sleep (QS) in both the prone and supine sleeping positions).	Supine position (active sleep and quiet sleep) (Daytime polysomnography to record the state of sleep).	Effects of sleeping position, sleep state and PNA on beat-beat heart rate (HR) and mean arterial pressure (MAP) responses with and without HUTs assessed during sleep in infants at 2-4 wks, 2-3 mo and 5-6 mo PNA.	Prone sleeping alters MAP responses to a HUT during QS at 2-3 mo PNA; tendency for BP to fall in the prone position appears to be prevented by elevated HR at 2-4 weeks and 5-6 mo, but not at 2-3 mo.	Two articles on the same study subjects published (considered as one study in this review); no information on allocation concealment or baseline imbalances.
54.	Yiallourou 2011 [70]	Monash university, Australia; HIC	Randomized crossover trial.	Full-term infants with normal birth weights and Apgar scores. Exclusion: Smoking mothers. N = 31. Gest = 40 wk/ BW = 3.6 kg.	Prone position (active sleep and quiet sleep) (Daytime polysomnography to record the state of sleep).	Supine position (active sleep and quiet sleep) (In each condition, three 1-2 min baseline measurements and three 15° head-up tilts were performed).	Baroreflex sensitivity (BRS) assessed using cross-spectral analysis (BRSSP) and sequence analysis (BRSSEQ) in the baseline condition and with BRSSP during head-up tilting (BRSSP Tilt).	Sleeping position, sleep state and postnatal age all affect infant baroreflex function. BRS is lower in QS, in the prone sleeping position and in earlier postnatal development.	Blood pressure and heart rate data needs to be free of movement artifact for accuracy of BRS assessment, which was difficult in active sleep.

BW – birth weight, ECG – electrocardiogram, Gest – gestation, HIC – high-income country, LMIC – lower middle-income country, OR – odds ratio, SIDS – sudden infant death syndrome, SUDI – Sudden Unexpected Death in Infancy, UMIC – upper middle -income country, y – year, mo – month, wk – week

### Design

The designs of the included studies were case-control (n = 28), cohort (n = 12), intervention trials (n = 11; including 6 crossover-randomized trials), ecological (n = 2) and survey (n = 1). The interventional trials evaluated physiological parameters in infants in university hospitals at one or several time points (see **Online Supplementary Document**).

### Setting

53 studies were conducted in high-income and one in an upper-middle income country (Brazil). All observational studies were based on data from the community, either from national health database, health surveys, or follow-up data from cohort studies.

### Participants

This review included data from 54 studies involving 474 672 participants, of which 49 studies with 80 974 participants were included in the quantitative analysis. Studies reporting SIDS, SUDI, or ALTE included infants up to 365 days of age, while studies reporting other outcomes involved participants who had their sleep position practices (exposure status) recorded as neonates. Though detailed population characteristics were not available in the case-control studies, available information suggests that 7%-13% of the participants were born before 37 weeks, with higher proportion of preterm infants among SIDS cases. There were no indications that the included infants were not healthy before the occurrence of SIDS, SUDI, or ALTE.

### Exposure status/ intervention (sleep position)

In case-control studies, data on sleep position were collected from records of death scene evaluation after SIDS, or through interviews at home visits, postal questionnaires and mail or phone surveys for control infants. The infant sleep positions (recorded in SIDS meta-analysis) were last sleep position (10 studies), usual sleep position (13 studies), and position last found at the death scene (2 studies). One study [36] did not mention the type of recorded sleep position.

In cohort studies, sleep position practices were prospectively recorded at one or multiple time points during follow-up. One study [60] used a mailed questionnaire to enquire about the usual infant sleeping position from 400 mothers in a survey. In ecological studies, the authors estimated the effect of sleep position looking at time-trends in SIDS and SUDI rates from national databases.

### Outcomes

None of the included studies reported the risk of neonatal mortality. The critical outcomes reported in the included studies were SIDS, SUDI, ALTE, and neurodevelopment outcomes (details in Appendix S2 in **Online Supplementary Document**).

SIDS was reported by 29 studies, 26 of which could be included in meta-analysis. These studies included SIDS cases reported in various infant death registries, national databases, or health department reports, from 1958 up to 2006. The definitions of SIDS in the studies were consistent with our protocol, recorded during the first year of life. Two studies reported deaths in children from 2 weeks to 2 years [26] and 2 weeks to 3 years [20]. We included these two studies because the mean age at the time of SIDS was 3-4 months, similar to other included SIDS studies. The controls were selected from hospital records, population databases, or randomly selected from the same geographical area and time as the cases. The control infants were matched for age, region, sex, and/or ethnicity in 17 studies.

### Risk of bias in included studies

A summary of the risk of bias assessment for 54 included studies is provided in Appendix S3 in the **Online Supplementary Document**. Of 48 observational studies, 46 were considered to be at serious risk of bias, mostly due to confounding and misclassification bias.

### Effects of interventions

A summary of the included studies’ effects on reported outcomes is shown in **Table 2**. The results on physiological parameters and studies with qualitative data have been summarized in Appendix S5, S6 in the **Online Supplementary Document**.

**Table 2:** Summary of studies: effect of sleep position on various outcomes by type of study design*

S. No.	Outcomes	Case-control	Intervention trials	Cohort	Ecological	Survey	All studies combined†
1.	SIDS	FS-15, FN-0, NE-9		FS-1, FN-0, NE-1	FS-2, FN-0, NE-0	FS-1, FN-0, NE-0	Favours supine sleep position
2.	SUDI	FS-1, FN-0, NE-0			FS-0, FN-0, NE-1		Limited evidence
3.	Unexplained SID/s-ALTE	FS-1, FN-0, NE-0					Limited evidence
4.	ALTE			FS-0, FN-0, NE-1			Limited evidence
5.	Short-term gross motor development (at 3-6 mo)			FS-0, FP-3, NE-1			Favours prone sleep position
6.	Long-term gross motor development (at 15-18 mo)			FS-0, FP-0, NE-2			No effect
7.	Fine motor development at 4-6 or 15-18 mo			FS-0, FP-0, NE-2			No effect
8.	Positional plagiocephaly	FS-0, FN-1, NE-0		FS-0, FN-4, NE-2			Favours non-supine sleep position
9.	Oxygen Saturation (SpO2)		FS-2, FP-0, NE-3				Limited evidence
10.	Cardiac output		FS-2, FP-0, NE-0				Limited evidence
11.	Cerebral oxygenation		FS-1, FP-0, NE-1				Limited evidence

ALTE – apparent life-threatening event; FS – favors supine; FN – favors non -supine; FP – favors prone; NE – no effect; SIDS – sudden infant death syndrome; SUDI – sudden unexpected death in infancy

*The numbers in the boxes against FS/FN/FP/NE indicate the number of studies in their respective categories which favored supine/ non-supine/ prone or showed no effect.

†The overall effect is based on subjective assessment of the following factors: results from all studies (including ecological studies and surveys), number of studies, their sample sizes and study quality (risk of bias).

**Figure 2:**
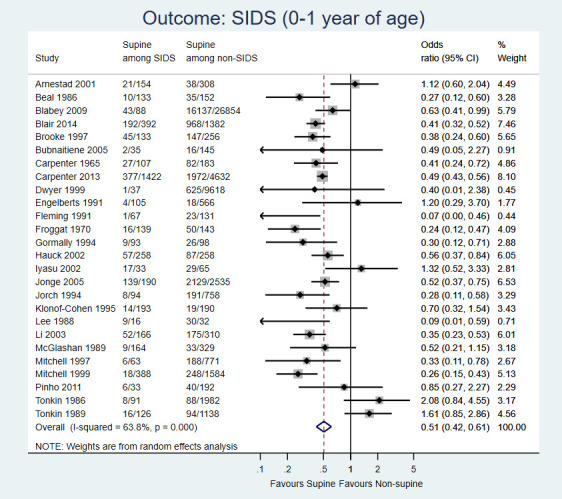
Forest plot for comparison: supine vs. non-supine sleep position. Outcome: SIDS (0-1 year).

26 observational studies, comprising 59 332 infants, could be pooled together in a meta-analysis for the outcome of SIDS (**Figure 2**). The meta-analysis favoured supine sleeping position (OR = 0.51, 95% CI = 0.42-0.61; very low certainty evidence) for the prevention of SIDS (0-1 year). Though there was substantial heterogeneity across the studies (*I ^2^* = 64%), the direction of the effects was mostly consistent. There was doubtful asymmetry in the funnel plot (**Figure S5** in **Online Supplementary Document**); however, the Egger and Begg tests for publication bias were not significant (lowest *P* = 0.79) for small-study effects. When compared to prone and side position separately, supine was protective for SIDS compared to prone (OR = 0.31, 95% CI = 0.21-0.45), but not to side position (OR = 0.80, 95% CI = 0.63-1.02), though the trend was still in favour of supine position (evidence not graded; Figures S6, S7 in the **Online Supplementary Document**).

One study reported the effect of sleep position on SUDI by comparing the last sleep positions in 126 cases of SUDI and 258 controls during 2012-2015 [62]. The unadjusted OR was reported to be 0.39 (95% CI = 0.23-0.65; low certainty evidence) for the prevention of SUDI, thereby favouring supine sleep position.

A case-control study reported on 29 newborns who suffered unexplained SID or severe-ALTE and 90 control newborns, with cases occurring within 24 hours of life in German hospitals [16]. The unadjusted OR was 0.16 (95% CI = 0.03-0.82; very low certainty evidence) for supine sleep position, compared to potentially asphyxiating position (defined in the study as infant lying on mother’s breast/abdomen or near to and facing her).

Another study [41] using data on 3729 infants, examined the effect of sleep position on hospital admission related to ALTE in their follow-up cohort of infants who slept consistently in prone, side, or supine position at 1, 3, and 6 months of age. The study did not report any difference (OR = 0.23, 95% CI = 0.005-2.04; very low certainty evidence) in the outcome among infants sleeping in supine position (1/1745), compared to non-supine (5/1984).

Another study followed-up a cohort of 14 138 infants delivered during 1991-92 in the United Kingdom to 18 months of age to assess the effect of sleep positioning on infant development [29]. The authors assessed various domains of development using the DDST at 6 and 18 months. The study reported the position effect as odds ratio by dichotomizing the gross motor scores using a -0.5 SD cut-off point after transforming the scores into a mean of zero and a standard deviation (SD) unit of 1. Based on data from 2097 participants, supine position significantly increased the odds of being 0.5 SD below mean on the Gross Motor Scale at 6 months, when compared to prone position (OR = 1.67, 95% CI = 1.22-2.27; moderate certainty evidence) but no difference when compared to side sleep position (OR = 1.02, 95% CI = 0.91-1.15; 8012 infants; low certainty evidence). The effect of the sleep position, however, diminished over time and was no longer significant at 18 months of age. The odds ratios for supine position being 0.5 SD below the mean on the Gross Motor Scale at 18 months were 1.16 (95% CI = 0.96-1.43; low certainty evidence) when compared to prone and 0.89 (95% CI = 0.69-1.16; low certainty evidence) when compared to side sleep position.

**Figure 3:**
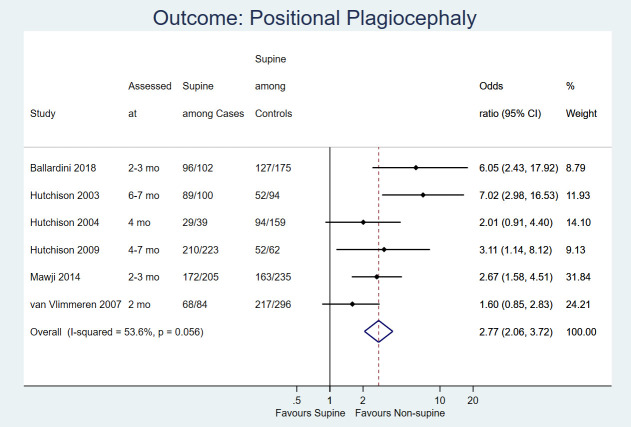
Forest plot for comparison: supine vs. non-supine sleep position. Outcome: Positional plagiocephaly.

Six observational studies reported the effect of sleep position on the occurrence of positional plagiocephaly at various ages (2-7 months; **Figure 3**). Supine position was associated with increased odds of positional plagiocephaly (OR = 2.77, 95% CI = 2.06-3.72; 1774 infants; *I^2^* = 53.6%; low certainty evidence) compared to non-supine sleep position.

## DISCUSSION

Low- to very low-certainty evidence suggested that supine sleep position might result in reduction of SUDI (0-1 year), SIDS (0-1 year), and unexplained SID/s-ALTE (neonatal period). The effect of supine sleep position was uncertain on ALTE-related hospital admissions in the first 6 months of life. Supine sleep position, compared to prone, likely increased the odds of being 0.5 SD below the mean on the Gross Motor Scale at 6 months, but the effect might not persist at 18 months. Supine, when compared to side sleep position, might not result in any difference in gross motor development (6 or 18 months). There might be an increase in the incidence of positional plagiocephaly (at 2-7 months of age) due to supine sleep position, compared to non-supine position. The evidence for most outcomes were of low- to very low-certainty, owing to high risk of bias (in observational studies), heterogeneity, and imprecision (where results were based on single study).

SIDS outcome was affected by significant heterogeneity (*I^2^* = 64%) partly attributable to disparate study designs, geographical locations (13 countries, 5 continents), and study periods (1958 to 2004), based on exploratory subgroup analyses (details in Appendix S4 in **Online Supplementary Document**). Safe sleep campaigns have been shown to impact SIDS and SUDI rates differently in various parts of the world [71]. Our review did not find an association between sleep position and ALTE-related hospital admissions. ALTE is considered to be a distinct entity from SIDS, and the SIDS prevention interventions have not made any significant impact on the occurrence of ALTEs [72]. The association of unexplained SID/s-ALTE episodes on the first day of life with potentially asphyxiating position was very uncertain, and reassuringly, increasing rates of skin-to-skin care have been temporally associated with decreasing SUDI prevalence in the first 6 days after birth in the US and Massachusetts [73].

Though development outcomes were evaluated by four included studies, the results could not be meta-analysed together due to variations in reported outcome measures. We presented data from a study by Dewey et al. [29] because this study had the largest number of participants with objective assessment scores (using Denver Developmental Screening Test) and was judged to be at moderate risk of bias (others were at serious risk of bias). Supine sleep position was found to increase the risk of positional plagiocephaly in our review. Positional plagiocephaly is considered benign and of mainly cosmetic concern, though some studies have associated its severity with poor neurodevelopment. These associations are thought to be marker of developmental risk rather than truly causal [74].

The findings of our review are in coherence with multiple infant sleep safety guidelines recommending supine sleep position for the prevention of SIDS [75]. One systematic review included 40 case-control studies on the association of infant sleep position and SIDS [9], 17 of which overlapped with our review. The other studies in the review were excluded either because they reported comparison of only ‘prone vs non-prone’ position without further qualification of non-prone position (16 studies) or their data sets were included in the more recent publications (seven studies) [9]. The results from that review [9] found increased odds of SIDS with prone and side sleep positions, compared to supine position. A review on factors affecting gross motor development (GMD) concluded that prone sleeping was associated with better GMD at 4 to 10 months but not at a later age (11 to 17 months), similar to our review [12]. Two studies from this review could not be included in our review because they did not record the exposure status (sleep position) in neonatal period.

This review aimed to reinforce the evidence on a safe sleep position for mortality and serious morbidities in term healthy neonates. We followed a rigorous methodology, with an all-inclusive literature search and no language filters. Given the rarity of SIDS and SUDI, we could not find any interventional studies which assessed the effect of intervention (sleep position) on these outcomes. Though a systematic review had looked at studies on interventions to reduce the risk of SIDS, it could only find studies evaluating the effectiveness in changing infant sleep practices, rather than the risk of SIDS itself [76]. We used manually calculated unadjusted unmatched ORs for pooling the results for the main comparison, since matched ORs were not reported separately for non-supine position in the studies. The sleep position was recorded differently across the studies. While last sleep position is the most likely to be related to SIDS risk, the usual and last found positions might be less accurate and therefore were least preferred for meta-analysis.

## CONCLUSIONS

Low- to very low-certainty evidence suggests that supine sleep position may reduce the risk of SIDS (0-1 year), SUDI (0-1 year), and unexplained SID/severe-ALTE (neonatal period), compared to non-supine position. There may be delay in short-term ‘gross motor’ development (6 months) and increased incidence of positional plagiocephaly (2-7 months) with prolonged supine sleep position, compared to prone, but the evidence suggests that it does not affect the long-term neurodevelopment (18 months). However, most reported outcomes in this review are limited by low- to very low-certainty evidence.

## Additional material


Online Supplementary Document
