# Supplementary material for: Effect of sleep position in term healthy newborns on sudden infant death syndrome and other infant outcomes: A systematic review
Source: J Glob Health. 2022 Jul 16;12:12001. doi: 10.7189/jogh.12.12001 (PMC9284601; doi:10.7189/jogh.12.12001)
Supplement: Online Supplementary Document [file jogh-12-12001-s001.pdf]

ONLINE SUPPLEMENTARY DOCUMENT

Effect of Sleep Position in Term Healthy Newborns on Sudden Infant Death Syndrome (SIDS) and Other Infant Outcomes: A Systematic Review

Appendix S1: Search strategy

We used the following search terms for MEDLINE: (Newborn OR infant OR neonat*) AND (sleep* OR position* OR posture OR prone OR supine). Similar terms were used for searching the other databases.

Appendix S2: Details of the outcomes reported in included studies

SUDI was reported by Mitchell 2017 and Muller-Nordhorn 2021, which by definition, included all SIDS cases along with explained cases in infants between 7 and 365 days of age. Hunt 2003 reported hospital admissions related to ALTE; however, the definition of ALTE was not clearly mentioned.

Poets 2012 studied the association between unexplained SID or severe-ALTE in term infants within 24 hours of birth and their sleeping positions. s-ALTE was defined as an acute state of cyanosis or pallor and unconsciousness, which was felt to require bagging, or intubation with or without cardiac compressions. We considered inappropriate to pool this outcome with other outcomes, since the outcome was distinct from SIDS or ALTE alone.

The association of infant sleep position with neurodevelopment outcomes was assessed by four studies (Davis 1998, Dewey 1998, Dwyer 1999, and Majnemer 2006).[1–4] Davis 1998 compared the mean ages of eight motor milestones attainment of prone and supine sleepers (recorded by parents until 6 months of age). Dewey 1998 compared the social, communication, fine and gross motor, and total developmental scale scores (based on the Denver Developmental Screening Test; DDST) at 6 and 18 months in infants sleeping prone, side, and supine. Dwyer 1999 derived the adjusted odds ratios for several motor and cognitive milestones (assessed as binary outcome with yes/no question) in infants at 12 weeks comparing different infant sleep positions. Majnemer 2006 compared the mean scores on Alberta Infant Motor Scale (AIMS) and Peabody Developmental Motor Scale (PDMS) at recruitment (4 and 6 months) and on PDMS and Battelle Developmental Inventory at 15 months follow up in prone and supine sleepers.[4]

Seven observational studies assessed plagiocephaly in infants with different tools: using Argenta's assessment tool at 2-3 months (Ballardini 2018; Mawji 2014), and visual and anthropometric examinations (Hutchison 2003, 2004, 2009 at 4-7 months; Leung 2017, van Vlimmeren 2007 at 2-3 months). We could not include data from Leung 2018 in meta-analysis because the study reported odds ratio of plagiocephaly for supine-sleep maximum (a continuous variable, defined as number of hours infant slept in supine position).[5]

Appendix S3: Risk of bias in included studies

Of 48 observational studies/ non-randomized trials, 46 were considered to be at serious risk of bias (**Figures S1 and S2**), mostly due to confounding and misclassification bias: 28 studies were at serious risk of bias for confounding because they either did not address the potential confounders (like maternal education, socio-economic status) or did not adjust for these confounders. Selection bias was serious in 15 studies either because the controls were not matched to cases for geographical area or age (10 studies) or the selection of participants could have related to outcome after exposure status (4 studies). 12 case-control studies were at serious risk of bias for misclassification of interventions due to recall bias expected in case interviews conducted remote to the event. 18 studies were at serious risk of bias for

deviation from intended interventions. These studies noted exposure status as usual sleeping position for "SIDS cases" and no information was available on adherence to the exposure status. All six randomized crossover trials were judged to be at some concern of bias owing to inadequate information on randomization process (**Figures S3 and S4**).

Appendix S4: Subgroup analyses for SIDS (0-1 year)

We explored the possible causes for significant heterogeneity noted for the outcome of SIDS (I^2=64%) by conducting subgroup analyses by study design, study period and location (**Figures S8, S9 and S10**). These causes could partly explain the heterogeneity.

Appendix S5: Effect of sleep position on physiological parameters

The intervention trials (n=11) evaluated the effect of infant sleep positions on respiration and hemodynamics in the neonatal period. These studies were mostly crossover in design; hence the same infant underwent supine and prone positioning in the sleep state, the latter being recorded either by daytime polysomnography or clinical observation.

Physiological parameters were reported by both randomized (n=6) and non-randomized (n=5) crossover studies. The participants in were term healthy neonates, except for Ma 2015, which included 9 preterm infants out of 35 participants.[6] One study (Lucchini 2015) provided p-values only and could not be included in the meta-analysis. Most of the studies compared supine with prone sleep position, except Poets 2009 which compared supine vs. side sleep position. All participants were studied in a well-controlled environment in medical universities ensuring their safety and accurately recording physiological parameters (by avoiding artefacts due to motion, arousals, body movements etc). Some trials also evaluated the effect of sleep state and postnatal age on these parameters by recording the outcomes in active sleep (AS) and quiet sleep (QS) in both the sleeping positions at various ages (2-4 weeks, 2-3 months and 5-6 months). The outcomes studied in these trials included stroke volume and cardiac output by electrical velocimetry, skin blood flow using Laser Doppler, systemic vascular resistance index, rate of desaturation events (<80%/h), minute ventilation and end-tidal carbon dioxide levels, arousal from sleep, heart rate (HR) responses following provoked arousal, HR responses to non-arousing trigeminal stimulation and baroreflex sensitivity.

Tables S1 and S2 list some of the important physiological parameters for the comparison of supine with prone sleep position in newborns. Most newborns were evaluated in quiet and active sleep states during 2-4 weeks of life, except three studies (Ma 2015, Rossor 2018, and Wu 2017) that had evaluated newborns in the first week of life. The studies reported mean values of the parameters and their standard deviations and had indicated if the differences were significant (p-value <0.05 for paired t-tests and repeated measures ANOVA). Since the study design was crossover, it was not possible to estimate the correlation factors and hence the effect sizes of the individual studies. Therefore, we could not pool these results.

Seven studies provided baseline heart rate in the neonates in supine and prone positions. Four studies reported significantly higher heart rate in prone sleep position, compared to supine position. There was no significant difference in Ma 2015 and Rossor 2018, while heart rate was higher in supine position in one study (Fister 2020; p-value >0.05). The increased heart rate in prone position may indicate a response to decreased stroke volume to maintain the cardiac output. Respiratory rate was not affected by sleep positions in two studies (Horne 2000 and Rossor 2018) but was significantly lower in prone position in one study (Fister 2020). Oxygen saturation was assessed with pulse oximetry in newborns in five studies - three of them showed no difference with sleep positions, while the other two studies (Fister 2020 and Wong 2019) reported significant lower SpO_2 in prone sleep position. Four studies evaluated the effect of

sleep positions on mean blood pressure (BP) by oscillometric method and three of them did not find any significant differences (Fister 2020, Ma 2015, and Yiallourou 2008). One study (Wong 2010) reported significantly higher mean BP values in prone sleep position, which might be a result of increased systemic vascular resistance.[6]

Two studies used electrical velocimetry to evaluate stroke volume and cardiac output in newborns in relation to sleep positions. Both studies (Ma 2015 and Wu 2017) reported significantly lower stroke volume as well as cardiac output in prone sleep position. Cerebral oxygenation ($CrSO_2$) was assessed by two studies with respect to sleep positions in term newborns. While Wong 2010 reported significantly higher $CrSO_2$ in supine position, compared to prone position, Wu 2017 did not report any difference between two positions.

Appendix S6: Results from studies with qualitative data

Katz et al evaluated the effect of residential altitude on SIDS rate in Colorado from 1990 to 2012 and noted a significant decrease in SIDS rate from 1.99 (1990-93) to 0.57 (1997-2012) per 1000 live births after the introduction of 'Back to Sleep' campaign in the US.[7] A survey by Mitchell 2007 found a substantial increase in the proportion of infants in New Zealand sleeping supine from 24% in 1992 to 72% in 2005, which could have accounted for 39%–48% decrease in SIDS mortality during this period.[8] Mitchell 2012 evaluated the impact of SIDS prevention campaign and calculated that over 3000 lives were saved over years (1990 to 2008) in New Zealand, and similarly, more than 17,000 and 40,000 lives were saved in England & Wales and the United States, respectively.[9] Muller-Nordhorn 2021 examined the relationship of immunization coverage and SUDI rates (1996–2015) using Poisson regression, adjusting for sleep position and poverty.[10] The authors reported crude and adjusted risk ratios of 0.90 (0.89-0.92) and 0.95 (0.90-1.00), respectively, for supine sleep position on the risk of SUDI, using multiple imputation due to missing data on sleep position.

References

1. Dwyer T, Ponsonby AL, Couper D, Cochrane J. Short-term morbidity and infant mortality among infants who slept supine at 1 month of age--a follow-up report. *Paediatr Perinat Epidemiol*. 1999;13(3):302-315. doi:10.1046/j.1365-3016.1999.00194.x

2. Davis BE, Moon RY, Sachs HC, Ottolini MC. Effects of sleep position on infant motor development. *Pediatrics*. 1998;102(5):1135-1140. doi:10.1542/peds.102.5.1135

3. Dewey C, Fleming P, Golding J. Does the supine sleeping position have any adverse effects on the child? II. Development in the first 18 months. ALSPAC Study Team. *Pediatrics*. 1998;101(1):E5. doi:10.1542/peds.101.1.e5

4. Majnemer A, Barr RG. Association between sleep position and early motor development. *J Pediatr*. 2006;149(5):623-629. doi:10.1016/j.jpeds.2006.05.009

5. Leung A, Mandrusiak A, Watter P, Gavranich J, Johnston LM. Impact of Parent Practices of Infant Positioning on Head Orientation Profile and Development of Positional Plagiocephaly in Healthy Term Infants. *Phys Occup Ther Pediatr*. 2018;38(1):1-14. doi:10.1080/01942638.2017.1287811

6. Ma M, Noori S, Maarek JM, Holschneider DP, Rubinstein EH, Seri I. Prone positioning decreases cardiac output and increases systemic vascular resistance in neonates. *J Perinatol Off J Calif Perinat Assoc*. 2015;35(6):424-427. doi:10.1038/jp.2014.230

7. Katz D, Shore S, Bandle B, Niermeyer S, Bol KA, Khanna A. Sudden infant death syndrome and residential altitude. *Pediatrics*. 2015;135(6):e1442-1449. doi:10.1542/peds.2014-2697

8. Mitchell EA, Hutchison L, Stewart AW. The continuing decline in SIDS mortality. *Arch Dis Child*. 2007;92(7):625-626. doi:10.1136/adc.2007.116194

9. Mitchell EA, Blair PS. SIDS prevention: 3000 lives saved but we can do better. *N Z Med J*. 2012;125(1359):50-57.

10. Müller-Nordhorn J, Neumann K, Keil T, Willich SN, Binting S. State-level trends in sudden unexpected infant death and immunization in the United States: an ecological study. *BMC Pediatr*. 2021;21(1):274. doi:10.1186/s12887-021-02733-w

Table S1. Comparison of important physiological parameters for supine vs. prone position in crossover studies*

Outcomes		Heart rate		Respiratory rate		Oxygen saturation (SpO_2)		Mean blood pressure	
Study	N	Supine	Prone	Supine	Prone	Supine	Prone	Supine	Prone
Fister 2020	46	137 (17)	130 (13)	47 (4)	45 (4)	97 (1.7)	96 (1.5)	51 (7)	54 (9)
Horne 2000	24	133 (2)	137 (2)	45 (2)	44 (3)	95 (0.2)	95 (0.3)		
Ma 2015	30	138 (17)	138 (18)					54 (7) (n=21)	57 (6) (n=21)
Rossor 2018	36	129 (10)	132 (10)	46 (12)	45 (11)				
Wong 2010	17	138 (2)	141 (2)			97 (0)	97 (0)	63 (2)	66 (2)
Wong 2019	17	123 (7)	129 (9)			98 (1.6)	97 (2.6)		
Wu 2017	34					98 (1.2)	98 (1.2)		
Yiallourou 2008	20	136 (3)	138 (2)					74 (4)	74 (4)

** The values provided in this table are means (standard deviations) for the given parameter. Unless specified, these values were obtained during quiet sleep in the studies.*

Table S2. Comparison of cerebral oxygenation and cardiac parameters for supine vs. prone position in crossover studies*

Outcomes		Cardiac output (EV) (ml/kg/min)		Stroke volume (EV) (ml/kg)		Cerebral oxygenation ($CrSO_2$)	
Study	N	Supine	Prone	Supine	Prone	Supine	Prone
Ma 2015	30	206 (44)	180 (41)	1.5 (0.3)	1.3 (0.3)		
Wong 2010	17	-	-	-	-	4% higher in supine ($p<0.05$)	
Wu 2017	34	182 (57)	170 (50)	1.46 (0.47)	1.36 (0.38)	75.5 (5.5)	76.4 (4.7)

** The values provided in this table are means (standard deviations) for the given parameter. Unless specified, these values were obtained during quiet sleep in the studies.*

EV- Electrical velocimetry

Table S3. Summary of findings table

Certainty assessment							№ of patients		Effect		Certainty (GRADE)	Importance
№ of studies	Study design	Risk of bias	Inconsistency	Indirectness	Imprecision	Other considerations	Sleeping in a supine position	Sleeping in a non-supine (prone or side) position	Relative (95% CI)	Absolute (95% CI)		
**Sudden infant death syndrome (SIDS; supine versus non-supine)**												
26	observational studies	very serious[a]	serious[b]	not serious	not serious	publication bias strongly suspected [c]	4720 cases 54612 controls		**OR 0.51** (0.42 to 0.61)	**48 fewer per 1,000** (from 58 fewer to 38 fewer)	⨁◯◯◯ VERY LOW	CRITICAL
**Sudden unexpected death in infancy (SUDI; supine versus non-supine)**												
1	observational study	very serious [d]	not serious	not serious	not serious	none	126 cases 258 controls		**OR 0.39** (0.23 to 0.65)	**219 fewer per 1,000** (from 313 fewer to 106 fewer)	⨁⨁◯◯ LOW	CRITICAL
**Unexplained SIDS or severe-ALTE (apparent life-threatening event) in neonatal period (supine versus non-supine)**												
1	observational study	very serious [a]	not serious	not serious	serious [e]	none	29 cases 90 controls		**OR 0.16** (0.03 to 0.82)	**232 fewer per 1,000** (from 282 fewer to 39 fewer)	⨁◯◯◯ VERY LOW	CRITICAL
**Gross motor development at 6 months (supine versus prone) (odds of being 0.5 SD below mean on the Gross Motor Scale) (assessed with: DDST at 6 months)**												
1	observational studies	serious [g]	not serious	not serious	not serious	none	-/1777	-/320	**OR 1.67** (1.22 to 2.27)	**0 fewer per 1,000** (from 0 fewer to 0 fewer)	⨁⨁⨁◯ MODERATE	CRITICAL
**Gross motor development at 6 months (supine versus side) (odds of being 0.5 SD below mean on the Gross Motor Scale) (assessed with: DDST at 6 months)**												
1	observational studies	serious [g]	not serious	not serious	Serious [h]	none	-/1777	-/6235	**OR 1.02** (0.91 to 1.15)	**0 fewer per 1,000** (from 0 fewer to 0 fewer)	⨁⨁◯◯ LOW	CRITICAL
**Gross motor development at 18 months supine versus prone) (odds of being 0.5 SD below mean on the Gross Motor Scale) (assessed with: DDST at 18 months)**												

Certainty assessment							№ of patients		Effect		Certainty (GRADE)	Importance
№ of studies	Study design	Risk of bias	Inconsistency	Indirectness	Imprecision	Other considerations	Sleeping in a supine position	Sleeping in a non-supine (prone or side) position	Relative (95% CI)	Absolute (95% CI)		
1	observational studies	serious [g]	not serious	not serious	serious [h]	none	-/1611	-/308	**OR 1.16** (0.96 to 1.43)	**0 fewer per 1,000** (from 0 fewer to 0 fewer)	⊕⊕◯◯ LOW	CRITICAL
**Gross motor development at 18 months (supine versus side) (odds of being 0.5 SD below mean on the Gross Motor Scale) (assessed with: DDST at 18 months)**												
1	observational studies	serious [g]	not serious	not serious	serious [h]	none	-/1611	-/5892	**OR 0.89** (0.69 to 1.16)	**0 fewer per 1,000** (from 0 fewer to 0 fewer)	⊕⊕◯◯ LOW	CRITICAL
**Hospital admissions related to ALTE (supine versus non-supine)**												
1	observational study	very serious [a]	not serious	not serious	very serious [f,h]	none	1/1745 (0.1%)	5/1984 (0.3%)	**OR 0.230** (0.005 to 2.040)	**2 fewer per 1,000** (from 3 fewer to 3 more)	⊕◯◯◯ VERY LOW	CRITICAL
**Positional plagiocephaly (supine versus non-supine)**												
6	observational studies	very serious [a]	not serious	not serious	not serious	none	753 cases 1021 controls		**OR 2.77** (2.06 to 3.72)	**219 more per 1,000** (from 148 more to 292 more)	⊕⊕◯◯ LOW	CRITICAL

CI: Confidence interval; **OR:** Odds ratio

*The quality of evidence was reviewed by a team of external methodologists.

Explanations

a. Most of the pooled effect provided by studies "C".
b. Statistical heterogeneity (I2≥60% or Chi2≥0.05).
c. Evident asymmetry in funnel plot
d. The included study used unadjusted OR and was considered as very serious risk of bias
e. Less than 300 newborns in continuous outcomes or less than 400 newborns in dichotomous outcomes.
f. Less than 30 events
g. The pooled effect provided by study "B".
h. Wide confidence interval crossing the line of no effect.

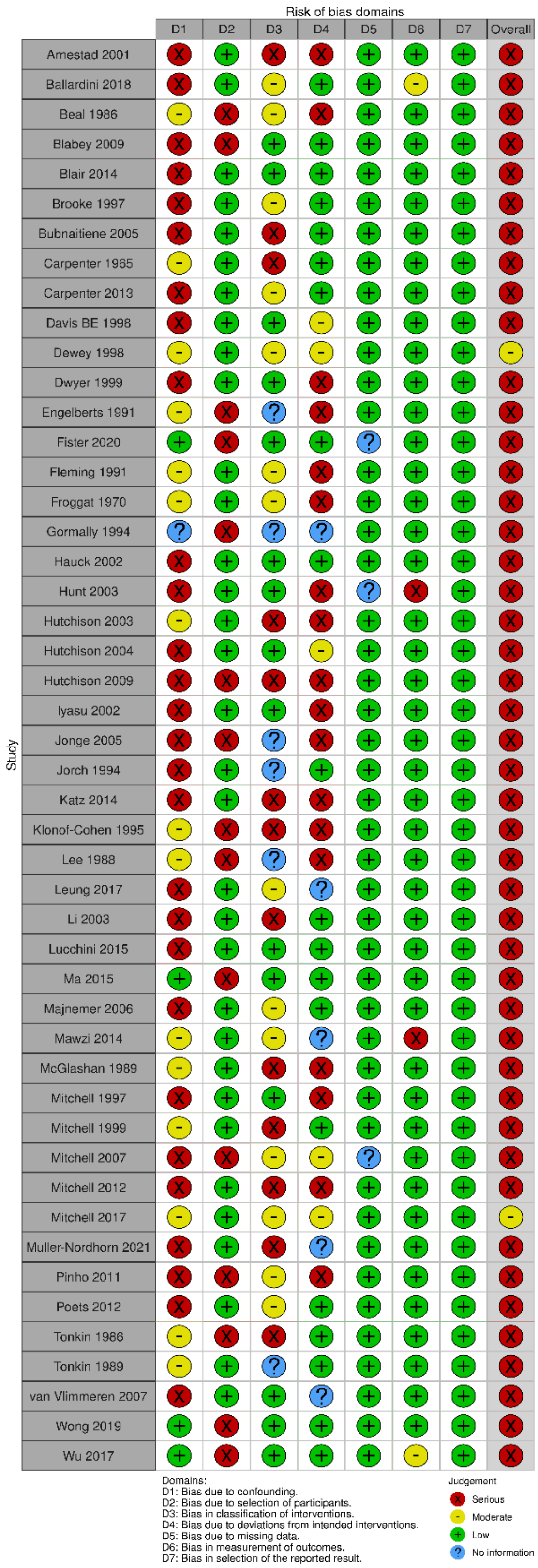

Figure S1. Risk of bias "traffic light" plots: review authors' judgements about each risk of bias item for each included study (observational studies)

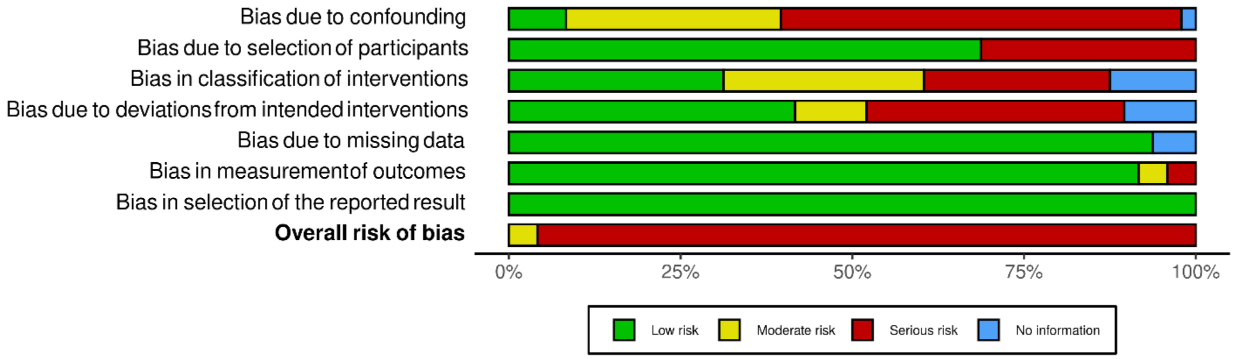

Figure S2. Risk of bias "weighted bar plots": review authors' judgements about each risk of bias item presented as percentages across all included studies (observational studies)

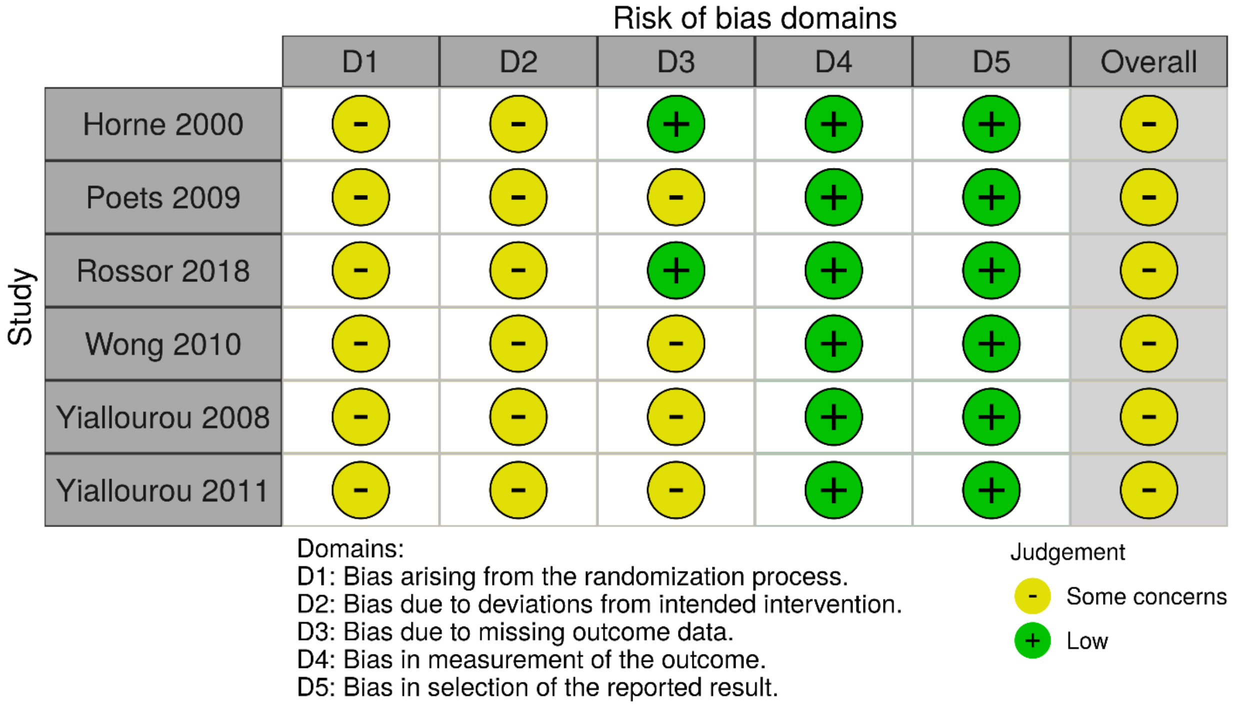

Figure S3. Risk of bias "traffic light" plots: review authors' judgements about each risk of bias item for each included study (randomized crossover trials)

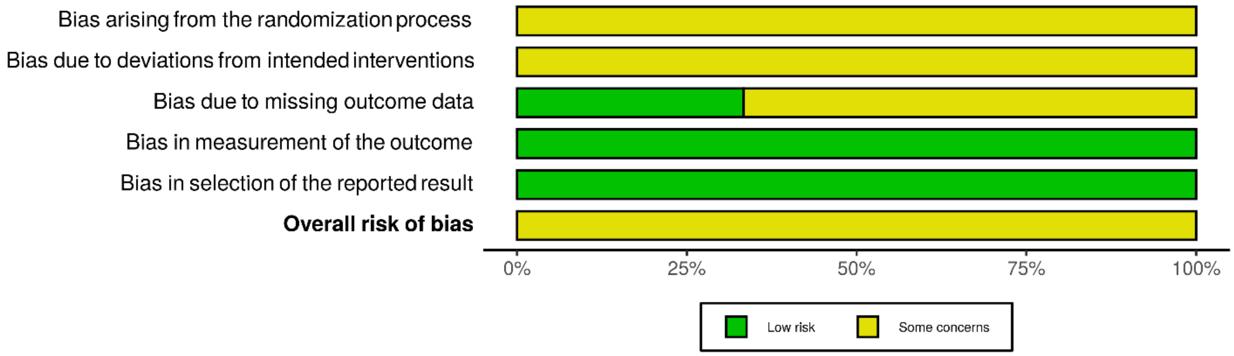

Figure S4. Risk of bias "weighted bar plots": review authors' judgements about each risk of bias item presented as percentages across all included studies (randomized crossover trials)

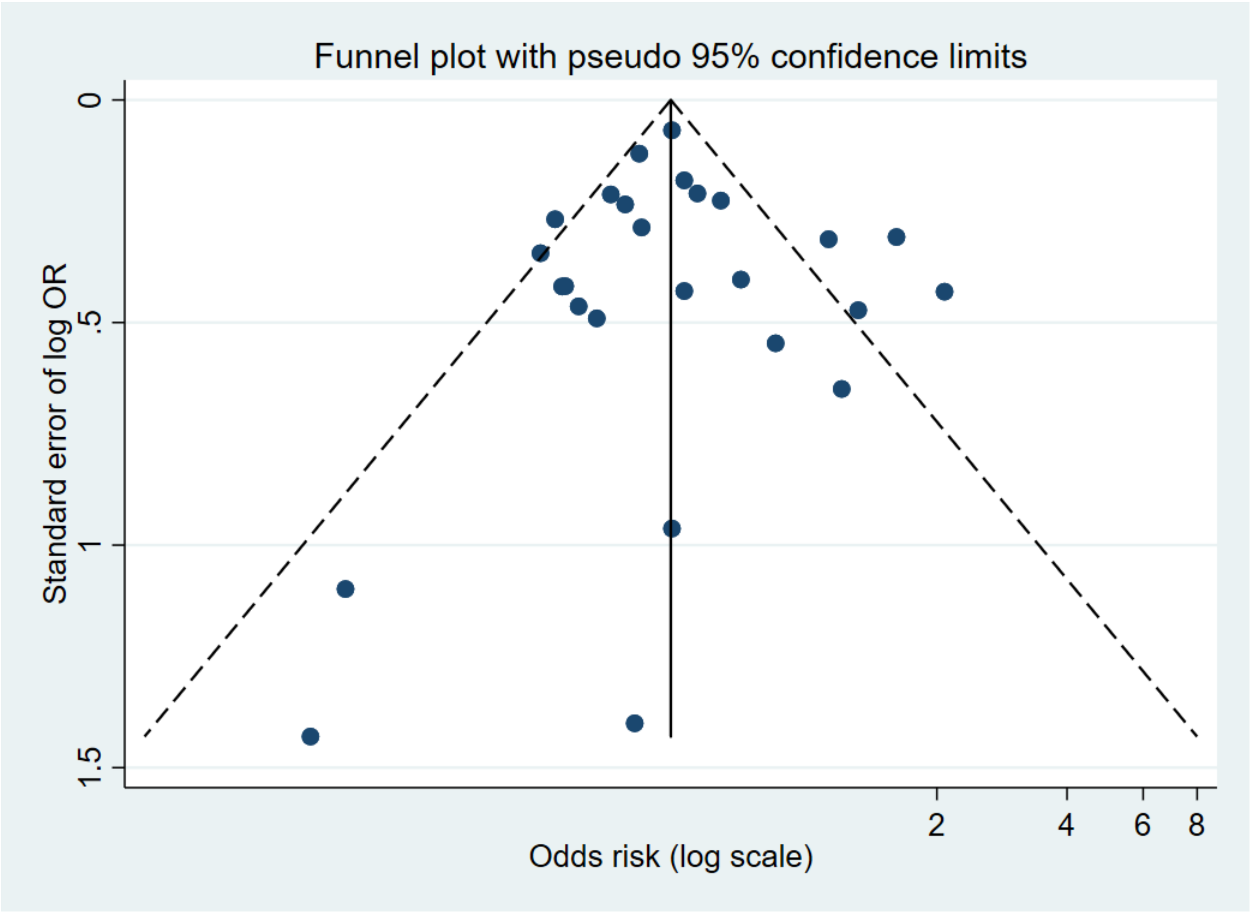

Figure S5. Funnel plot for comparison of SIDS (0-1 year) for supine vs. non-supine sleep position

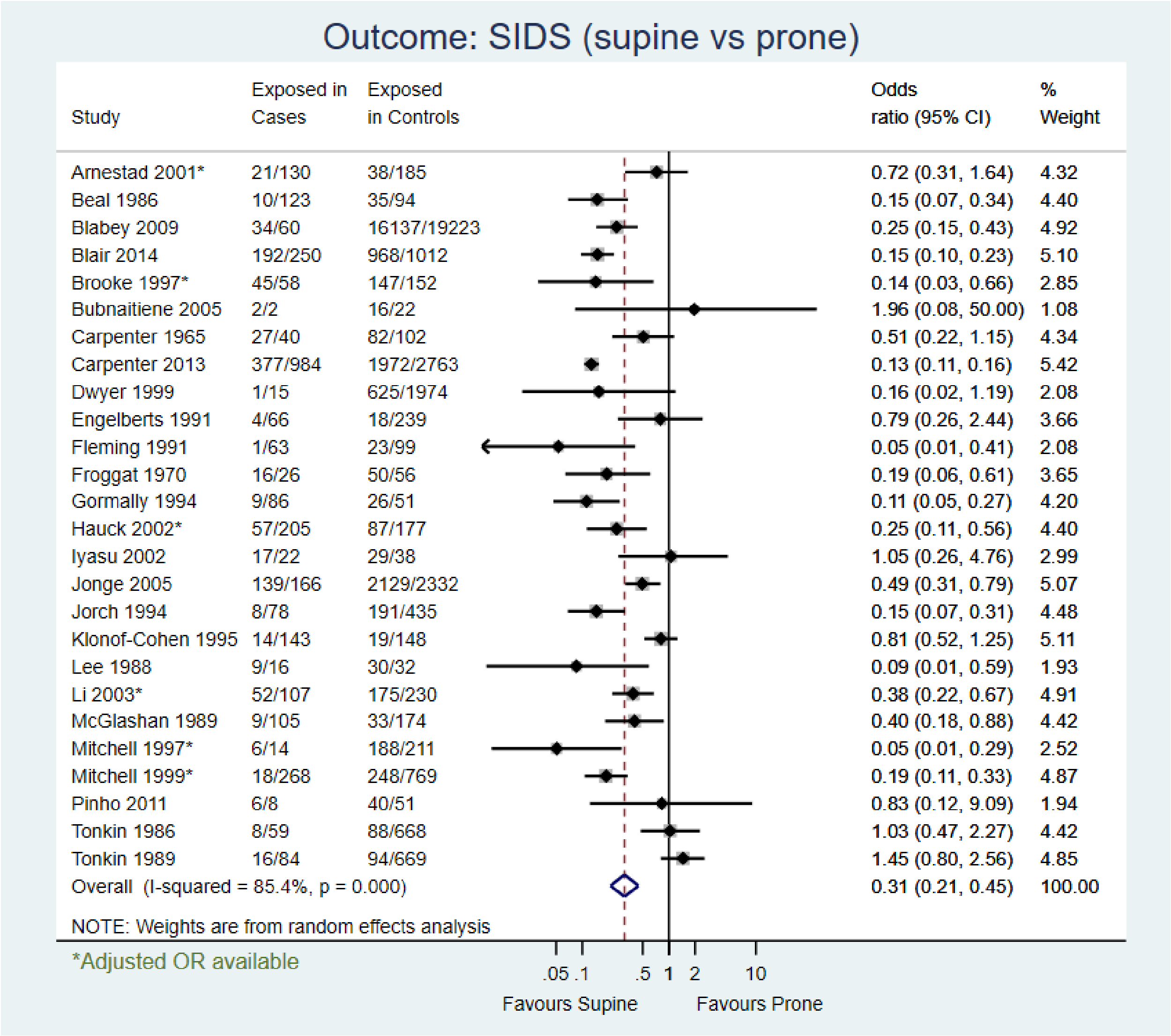

Figure S6. Comparison of SIDS (0-1 year) for supine vs. prone sleep position

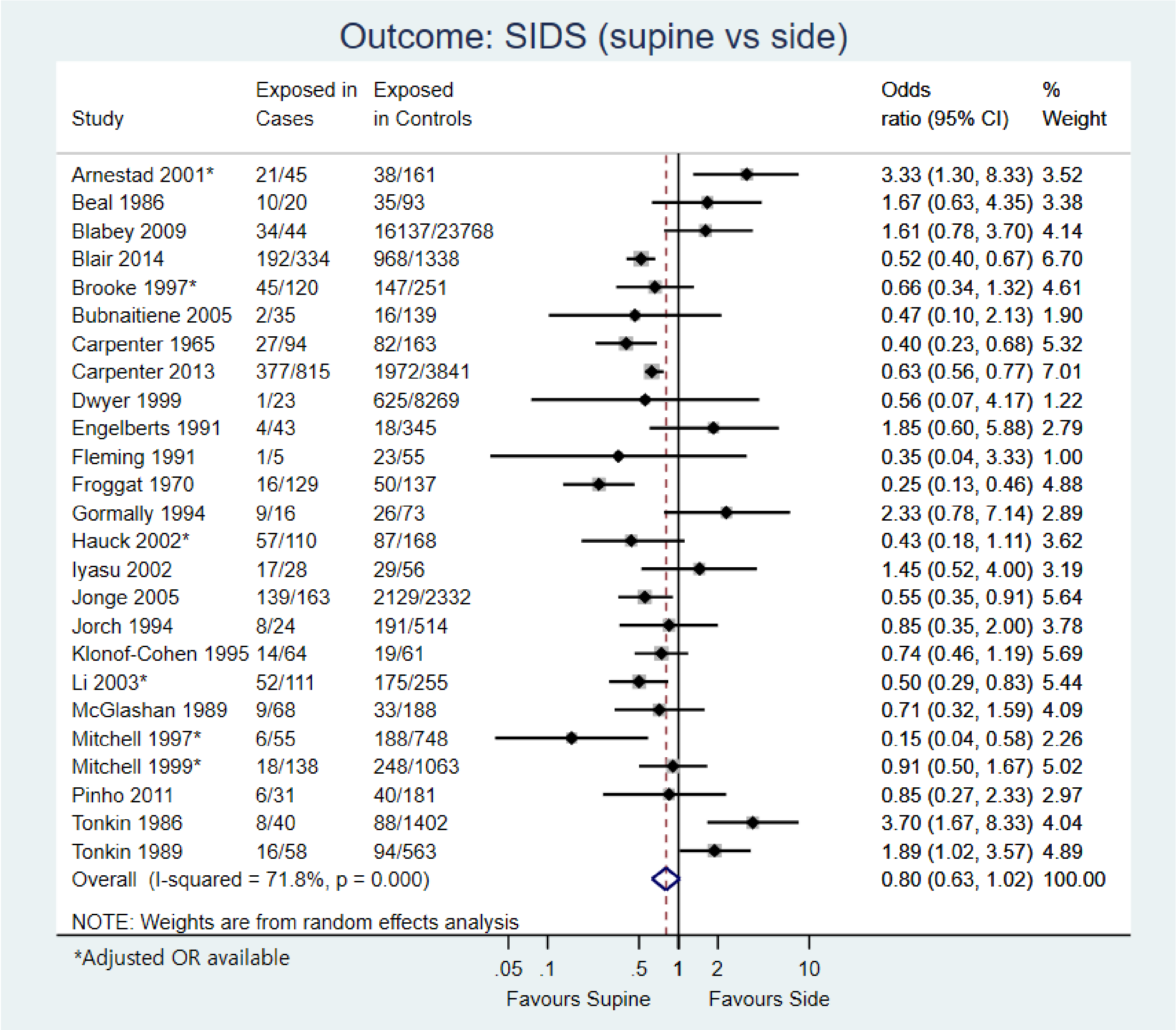

Figure S7. Comparison of SIDS (0-1 year) for supine vs. side sleep position

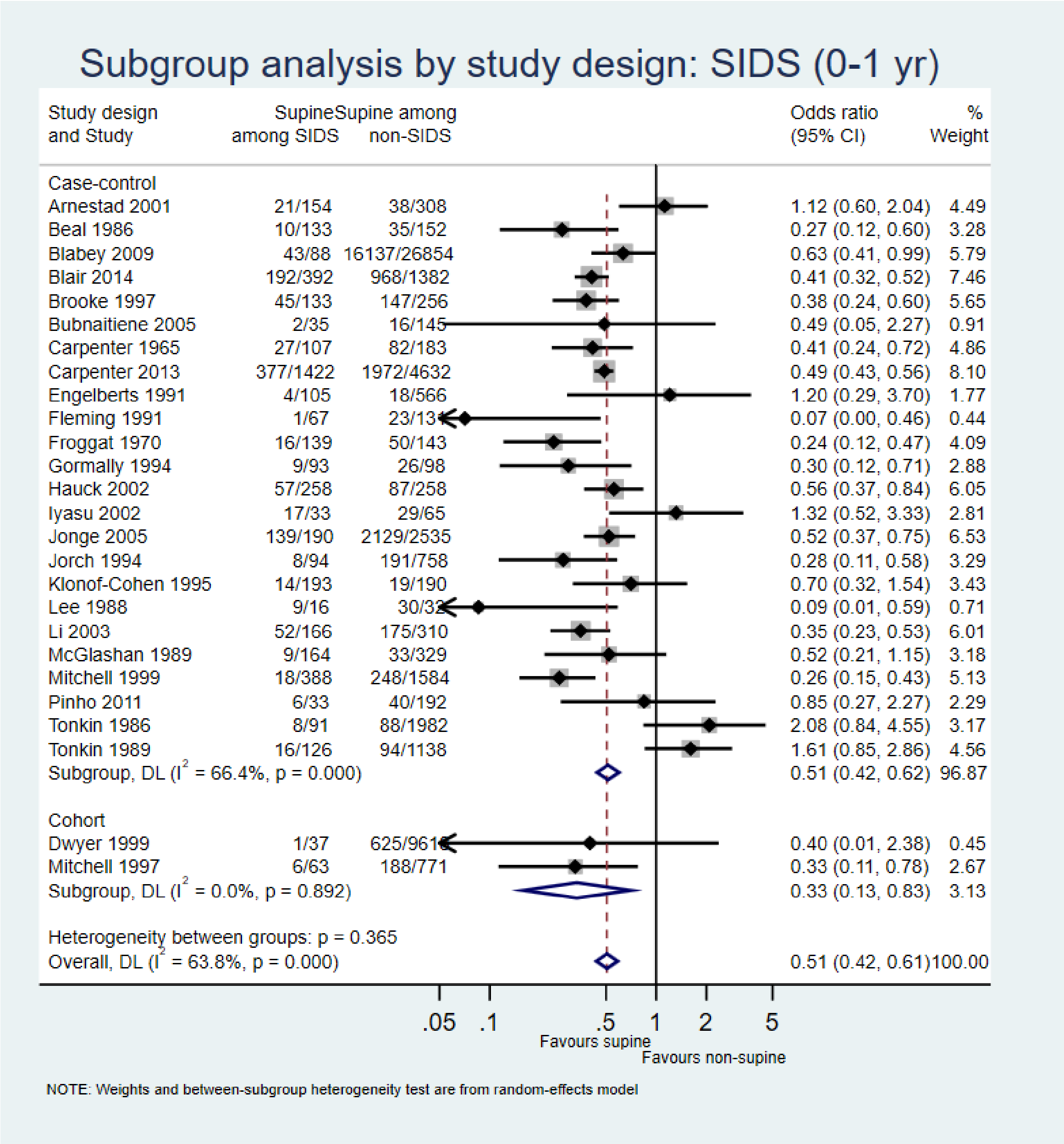

Figure S8. Comparison of SIDS (0-1 year) for supine vs. non-supine sleep position: Subgroup analysis by study design

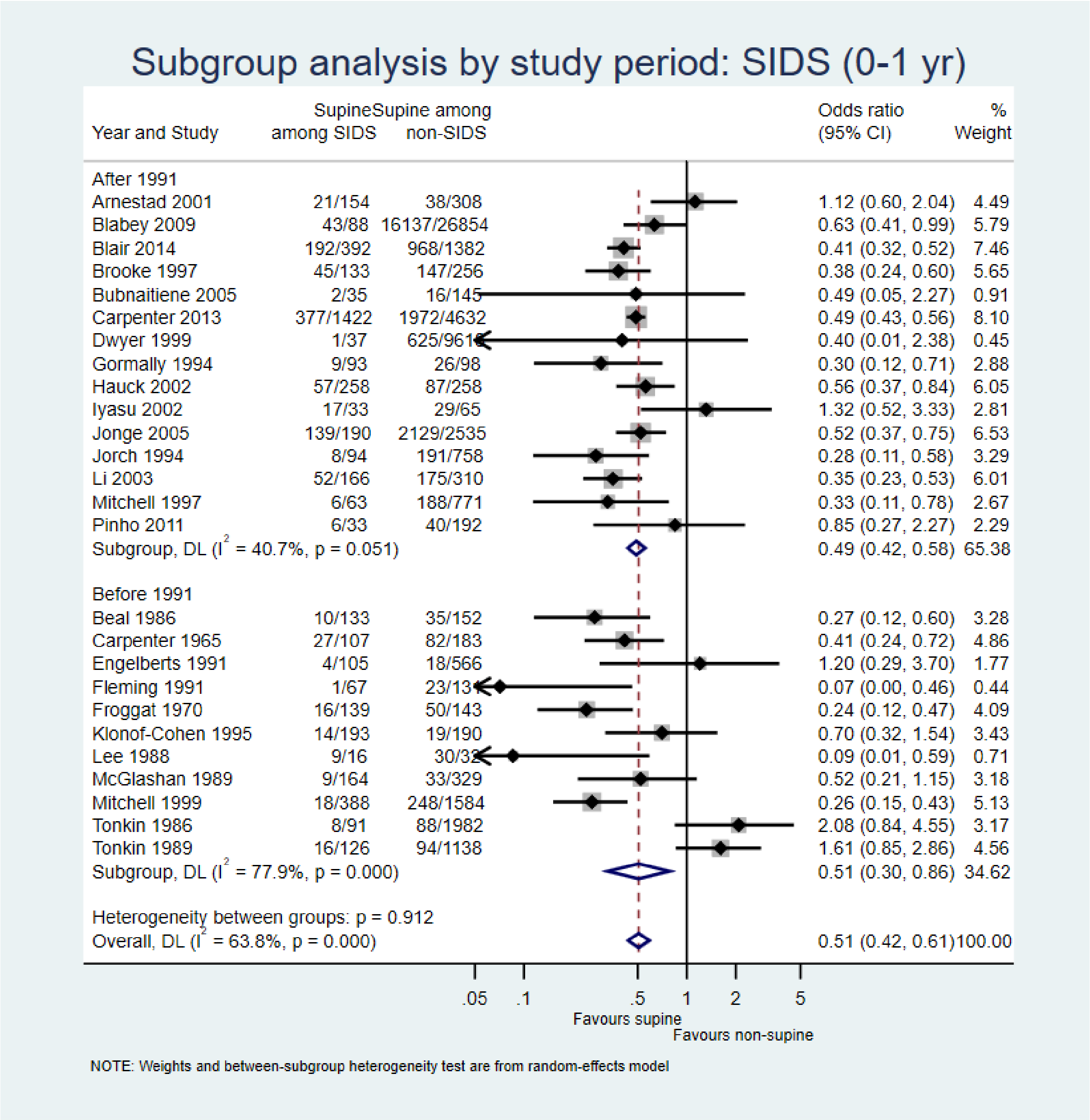

Figure S9. Comparison of SIDS (0-1 year) for supine vs. non-supine sleep position: Subgroup analysis by study period

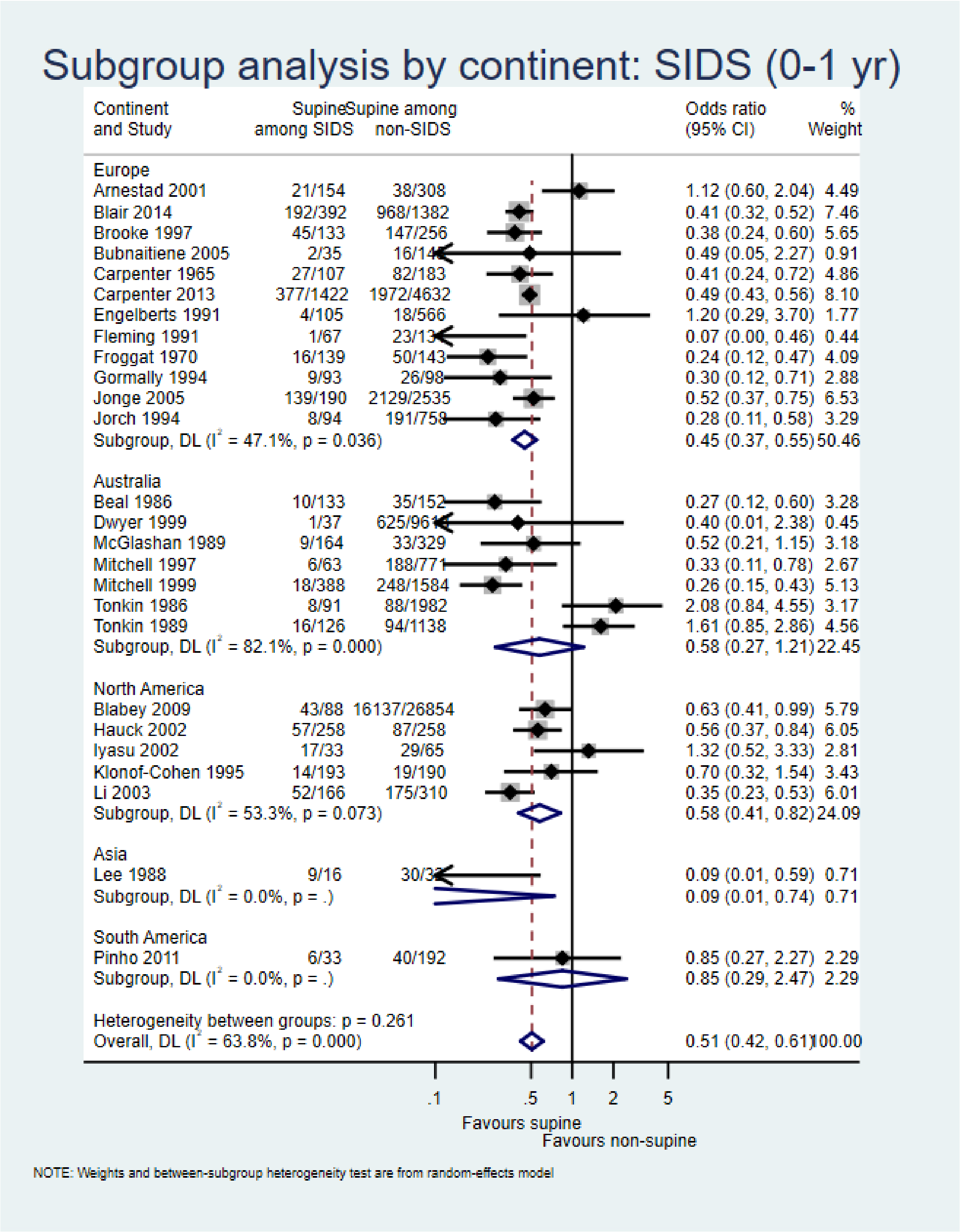

Figure S10. Comparison of SIDS (0-1 year) for supine vs. non-supine sleep position: Subgroup analysis by location (continent)
